# Gasdermins in neurodegeneration: emerging mechanisms and therapeutic targets

**DOI:** 10.1038/s41419-025-08373-7

**Published:** 2025-12-23

**Authors:** Sizhuo Chen, Jing Zhang, Xin Chen, Zhongmeng Lai, Zhenhuan Zhao, Shao-bin Wang

**Affiliations:** 1 National “111” Center for Cellular Regulation and Molecular Pharmaceutics, School of Life and Health Sciences, Hubei University of Technology, Wuhan, China; 2 Center for Advanced Vision Science, University of Virginia, Charlottesville, VA USA; 3 Department of Ophthalmology, University of Virginia, Charlottesville, VA USA; 4 Department of Anesthesiology, Fujian Medical University Union Hospital, Fuzhou, Fujian China

**Keywords:** Cell death in the nervous system, Neurodegenerative diseases

## Abstract

Gasdermins (GSDM) are pore-forming proteins that mediate pyroptosis, an inflammatory form of programmed cell death characterized by membrane permeabilization and the release of intracellular contents. Beyond their roles in host defense and immunity, recent studies have revealed critical contributions of GSDMs, particularly GSDMD and GSDME, to the pathogenesis of neurodegenerative disorders. Their functional scope has now expanded beyond executing cell death to roles in tissue regeneration and food tolerance. The recent discovery that intact, full-length GSDMs can form pores is prompting a reevaluation of long-standing models of gasdermin activation. How post-transcriptional modifications (PTMs) regulate this unconventional activity, and under what physiological or pathological contexts these alternative mechanisms are engaged, remains an open question. Moreover, the development of neutralizing biologics that specifically target GSDM pores opens new avenues for therapeutic intervention. In light of these emerging insights, this review will provide a comprehensive and up-to-date overview of recent breakthroughs in GSDM research. We highlight advances in the structural basis of GSDM activation and pore assembly. We also discuss how these mechanisms are involved in the pathogenesis of neurodegenerative diseases and therapeutic strategies based on the emerging small-molecule inhibitors and neutralizing biologics.

## Facts

- Activation of GSDME and GSDMD has been detected in neurons and microglia in ALS, MS, AD, and Parkinson’s disease, and blocking their activity has been shown to protect neurons and reduce neuroinflammation.
- Both cleavage-dependent and cleavage-independent mechanisms regulate GSDM activation and pore formation. Cleavage-independent GSDMs (e.g., *Tricho*GSDM, RCD-1) show conserved pore-forming architecture and may represent alternative activation pathways.
- Cysteine-targeting compounds and oligomerization blockers disrupt gasdermin assembly, which have shown potential to alleviate neuronal pyroptosis, and neuroinflammation in preclinical models of neurodegeneration.

## Open questions

- Which molecular determinants, such as lipid composition and subcellular localization, govern the balance between cleavage-dependent and cleavage-independent GSDM activation under physiological and pathological conditions?
- How do distinct GSDMs, acting in specific neural and immune cell types, coordinate the initiation, propagation, and chronic progression of neurodegenerative diseases?
- How do neurodegeneration-specific triggers (protein aggregation, mitochondrial dysfunction, and excitotoxic stress) modulate gasdermin activation in the CNS?
- Do gasdermin pores influence CNS-specific processes such as synaptic function and neuron–glia communication, thereby contributing to disease progression?
- Can GSDM inhibitors be used safely in humans for neurodegenerative diseases, or would systemic blockade disrupt indispensable physiological functions?

## Introduction

Pyroptosis represents a distinct form of programmed cell death that functionally differs from apoptosis and necroptosis [1, 2]. It is characterized by plasma membrane pore formation and the release of intracellular contents, triggering a potent inflammatory response [2]. Like necroptosis, pyroptosis serves as a host defense mechanism by promoting the release of immunogenic molecules, including damage-associated molecular patterns (DAMPs) and pathogen-associated molecular patterns (PAMPs) [3]. However, necroptosis is caspase-independent and driven by receptor-interacting protein kinases (RIPK1 and RIPK3) and the executioner mixed lineage kinase domain-like protein (MLKL) [4]. Pyroptosis is largely caspase-dependent, mediated by inflammatory caspases (such as caspase-1, -4, -5 in humans and caspase-11 in mice), and executed by members of the Gasdermins (GSDM) family of pore-forming proteins [2, 5, 6].

Gasdermin homologs are broadly conserved among eukaryotes [7]. In resting cells, GSDMs are maintained in an inactive state through autoinhibition by its C-terminal domain (CTD). Cleavage by inflammatory caspases removes this inhibition, enabling the N-terminal fragment to oligomerize and form large membrane pores [5, 8]. GSDM pores not only induce lytic cell death but also facilitate the release of cytokines and mitochondrial DNA, contributing to inflammation, immunity and tissue damage, implicated in a variety of human diseases, including neurodegenerative disorders [9–12]. In bacteria, GSDMs are regulated by a short ~25-residue C-terminal peptide rather than a well-folded inhibitory domain [13]. In early metazoans such as *Trichoplax adhaerens*, GSDMs consist only of a pore-forming domain that switches between monomeric and homodimer states through disulfide bond regulation [14]. The human GSDM family includes six homologs: GSDMA, GSDMB, GSDMC, GSDMD, GSDME (also known as DFNA5), and PJVK (also known as DFNB59). Human GSDM activations are generally cleavage-dependent. However, in some contexts, point mutations or post-translational modifications (PTMs) in the N-terminal domain can bypass the C-terminal mediated autoinhibition and induce pore formation by the full-length protein [15, 16]. The mechanisms underlying these alternative, cleavage-independent activation pathways are not yet fully defined.

## Regulation of gasdermin activation

Gasdermins first appeared as a single gene early in metazoan evolution [7, 17]. In the common ancestor of vertebrates, this gene underwent multiple duplication events, leading to the expansion of the gasdermin family, which reached its peak diversity in mammals. Gasdermin activity is regulated at multiple layers: transcription, RNA editing and splicing, post‑translational modifications, and proteolytic cleavage by caspases and granzymes.

### Gasdermin A

**Fig. 1:**
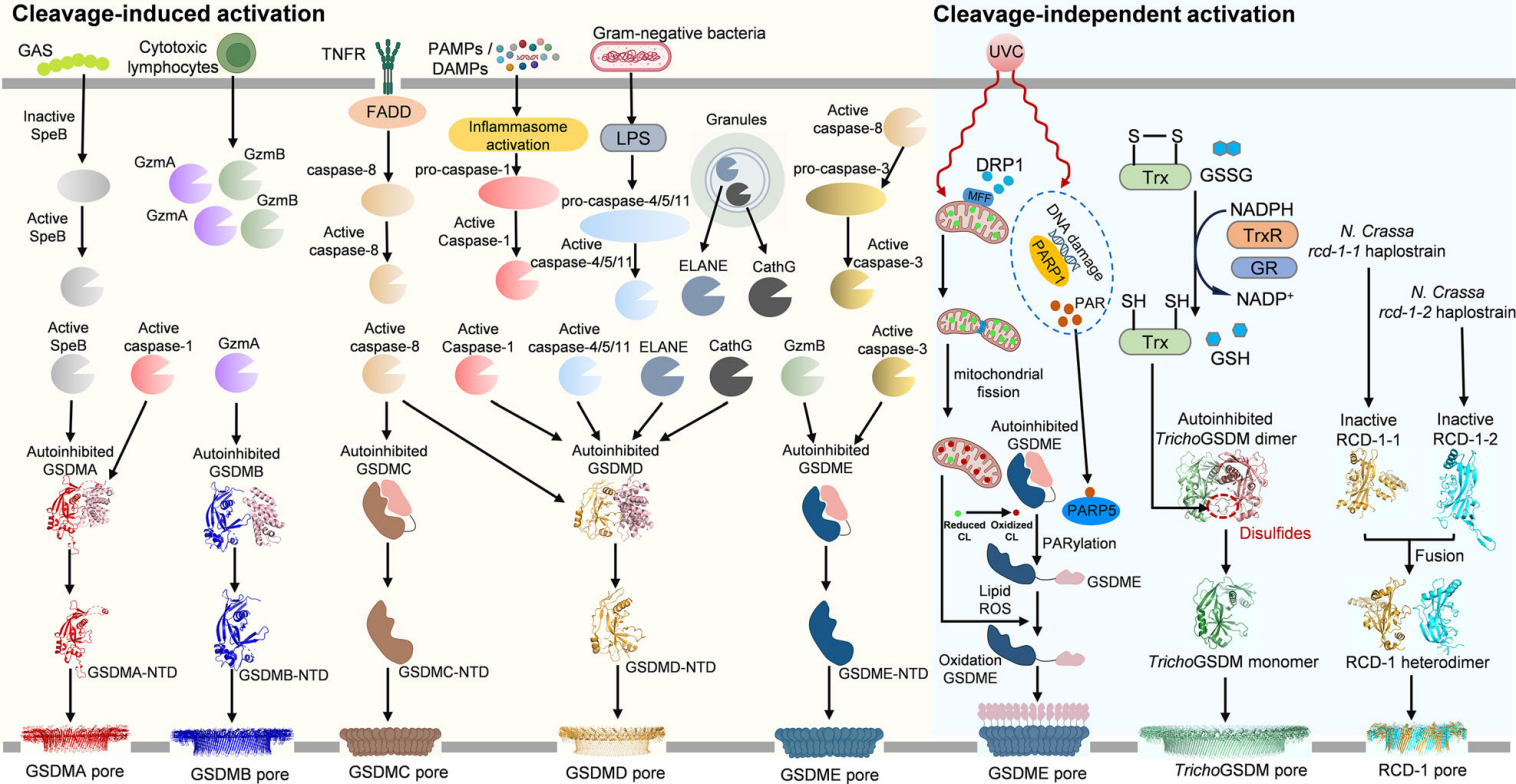
Canonical and non‑canonical pathways for gasdermin activation. Cleavage-dependent pathways (left) activate GSDMA, GSDMB, GSDMC, GSDMD and GSDME via bacterial SpeB, granzymes A/B, caspase‑3/8 or inflammasome associated caspase‑1/4/5/11; neutrophil elastase and cathepsin G also cleave GSDMD. Cleavage‑independent pathways (right) include ROS‑enhanced S-palmitoylation of GSDMD, PARylation of GSDME, oxidative stresses-driven monomerization of *Tricho*GSDM, and RCD‑1 heterodimer formation. Created with BioRender.com.

GSDMA was the first gasdermin isoform to be identified and the first whose pore structure was solved by cryo‑EM [18, 19]. It was initially discovered in mouse skin, where mutations at the Rim3 locus cause defective skin and hair development [20, 21]. In humans, GSDMA expression is largely restricted to epithelial cells of esophagus, bladder, and skin, where it plays a critical role in guarding against pathogen invasion. During Group A *Streptococcus* (GAS) infection [22, 23], the bacterial cysteine protease *SpeB* is activated and directly cleaves GSDMA at Gln246, leading to pyroptosis and local inflammation for bacterial clearance (Fig. 1). Of note, mammalian GSDMA lacks a functional caspase-1 cleavage site, rendering it resistant to canonical inflammasome-mediated activation. However, GSDMA orthologs in non-mammalian species, including birds, amphibians, and reptiles, retain a conserved caspase-1 cleavage motif and can be activated through caspase-mediated pathways [24].

### Gasdermin B

GSDMB shares sequence homology with GSDMA and is located within the gene clusters at 17q21 [25]. Notably, there are no *Gsdmb* orthologs found in rodents. Unlike GSDMA, which has a more restricted expression pattern, GSDMB is expressed broadly across multiple tissues, including the esophagus, airway epithelium, stomach, liver, small intestine and various immune cells. There are five isoforms of GSDMB derived from alternative splicing events, although the tissue- or cell-type specificity of each isoform remains incompletely understood [26]. GSDMB is not activated through cleavage by cellular caspases; instead, during Shigella flexneri infection it is targeted by the bacterial effector IpaH7.8 for ubiquitination and proteasomal degradation, while its pore-forming N-terminal fragment can be generated by granzyme A (GzmA) in antitumor immunity [27–29] (Fig. 1). GSDMB is indispensable for epithelial restitution and repair independent of pyroptosis [30]. In inflammatory bowel disease (IBD), GSDMB is upregulated in inflamed lesions and regulates intestinal epithelial cells (IEC) proliferation and migration for tissue repair. IBD-associated mutations in GSDMB dysregulated epithelial function. Additionally, polymorphisms in GSDMB have been strongly associated with increased susceptibility to chronic inflammatory diseases such as asthma and type 1 diabetes [31, 32].

### Gasdermin C

GSDMC (originally named MLZE, melanoma-derived leucine zipper, extra-nuclear factor) was first cloned from human melanoma cells [33, 34]. Its expression increases with melanoma progression and is similarly elevated in lung adenocarcinoma, correlating with poor clinical outcomes [35, 36]. Today, GSDMC has been found expressed in various tissues, including trachea, esophagus, spleen, small intestine, colon, and skin. In breast cancer cells, GSDMC expression is regulated by PD-L1, and specifically cleaved by caspase-8 in TNFα-induced tumor necrosis and α-ketoglutarate (α-KG)-induced tumor suppression [37, 38]. In anti-helminth immunity, GSDMC is cleaved by Cathepsin S and releases its N-terminal domain, which preferentially localizes with Rab7+ vesicles than plasma membrane. Activation of GSDMC impairs endosomal function, disrupting lipid droplet degradation and recycling, and reducing the synthesis of lipid-based immune mediators such as prostaglandin D2 (PGD2). Because PGD2 normally suppresses type II immune responses, its reduction enhances this response and promotes the clearance of intestinal parasitic infections in a pyroptosis-independent manner [39].

### Gasdermin D

GSDMD is the most extensively studied gasdermin and the primary executor of pyroptosis, playing a central role in a broad spectrum of inflammatory diseases [6, 40]. It can be activated by various proteases in response to PAMPs and DAMPs in myeloid, epithelial and endothelial cells (Fig. 1). Canonical inflammasome signaling activates GSDMD via caspase-1, whereas the non-canonical pathway involves direct cleavage of GSDMD by caspase-4 and -5 in humans (or caspase-11 in mice) following lipopolysaccharide (LPS) recognition [41–44]. Additionally, during *Yersinia* infection, macrophages can trigger a RIPK1–caspase-8–GSDMD pathway, where caspase-8 cleaves GSDMD to initiate pyroptosis and limit bacterial spread [45, 46]. In aged neutrophil, GSDMD can be cleaved by neutrophil elastase (ELANE) and cathepsin G (CathG) independently of caspases for lytic neutrophil deaths [43, 47–49]. In IECs, dietary antigens trigger caspase‑3/7–dependent generation of a non‑pyroptotic ~13‑kDa GSDMD fragment that translocates to the nucleus. This nuclear fragment upregulates CIITA and MHC II expression, sustaining type 1 regulatory T (Tr1) cells and maintaining oral tolerance to dietary antigens [50]. Additionally, a recent study reveals that reactive oxygen species (ROS)–driven S-palmitoylation of Cys191 enables GSDMD to form membrane pores in macrophage and monocytes even in the absence of proteolytic cleavage [16]. Inflammasome activation or pharmacological elevation of ROS increases the expression of ZDHHC5/9 palmitoyl transferases, which catalyze the addition of a palmitate moiety to Cys191/ Cys192 on both full-length and cleaved GSDMD. This modification promotes GSDMD membrane localization, oligomerization, and pore formation [51, 52]. In LPS-induced sepsis, blocking GSMD palmitoylation, either with the broad palmitoylation inhibitor 2-bromopalmitate or with GSDMD palmitoylation–specific competitive peptide (CPP-W) significantly alleviates systemic inflammation and improves survival [16, 52]. Notably, other human gasdermins, such as GSDME, are also palmitoylated at analogous N-terminal cysteines, suggesting that palmitoylation may serve as a conserved, family-wide regulatory checkpoint. However, the upstream palmitoyl transferase(s) responsible for GSDMD modification remain debated: one study identified DHHC7 as the primary enzyme [51], whereas others have implicated ZDHHC5 and ZDHHC9 [16, 52]. Moreover, upstream inflammasome components such as NLRP3 are palmitoylated by ZDHHC5/7 [53–55], raising the possibility that the observed effects of palmitoylation blockade may reflect modulation of upstream regulators rather than direct GSDMD palmitoylation. Beyond palmitoylation, GSDMD-mediated pyroptosis is also modulated by other PTMs, including ubiquitination [56], succination [57], phosphorylation [58], acetylation [59], and SUMOylation [60], which fine-tune gasdermin stability, localization, and pore-forming activity.

### Gasdermin E

GSDME, also known as deafness, autosomal dominant, 5 (DFNA5), was initially considered related to hereditary hearing loss [61, 62]. It was later found to share sequence similarity with other gasdermin family members, acting as a crucial regulator of apoptosis and pyroptosis [63, 64]. In Hela cells, GSDME expression levels determines the model of chemotherapy drug-induced cell death: GSDME-negative cells undergo classical apoptosis, whereas GSDME-high cells rapidly switch to pyroptosis. In cells with low GSDME expression, cell death exhibits a mixed phenotype with initial apoptotic morphology followed by the formation of pyroptotic bubbles and plasma membrane lysis [65, 66]. Moreover, in the absence or dysfunction of GSDMD, caspase-1 can induce GSDME cleavage and GSDME-dependent secondary necrosis/pyroptosis in cortical neurons and mast cells [67–69]. GSDME can also be activated by granzyme B (GzmB), a cytotoxic protease released by cytotoxic T lymphocytes and natural killer (NK) cells in antitumor immunity [70]. Recent work shows that GSDME can mediate cleavage‑independent pyroptosis (Fig. 1). Under ultraviolet (UV)-C radiation, DNA damage activates nuclear PARP1 and poly (ADP-ribose) (PAR) polymers release, which drive cytoplasmic PARP5‑dependent PARylation of GSDME [15]. The PARylated protein is further oxidated by lipid ROS for promoting full-length GSDME oligomerization, plasma membrane pore formation and pyroptosis. In addition to the plasma membrane, mitochondria is a major site of action for several gasdermins. In macrophages, cleaved GSDMD binds cardiolipin-enriched mitochondrial membranes, causing outer membrane permeabilization and cytochrome c release prior to plasma membrane damage. This mitochondrial damage has been shown to be required for the full execution of gasdermin-mediated pyroptosis [10]. Although direct evidence for GSDME–cardiolipin binding is still lacking, GSDME rapidly translocates to neuronal mitochondria, causing mitochondrial damage, neurite loss and neuron death preceding cell death [11].

### Pejvakin

Pejvakin protein, encoded by the PJVK gene, is a distant member of the GSDM family, and is widely expressed in various tissues including the kidney, lung, inner ear, liver, and with particularly high expression in the testis [71]. PJVK is structurally distinct from other GSDM proteins due to its truncated CTD and the absence of a linker region. The N-terminal domain of PJVK does not exhibit pore-forming activity [7]. Nonetheless, recent studies suggest that PJVK plays a protective role in auditory function [72, 73]. It has been shown to sense sound-induced elevations in ROS and facilitates LC3B-mediated autophagic degradation of peroxisomes, thereby helping to protect auditory hair cells from noise-induced damage [72].

## Structure basis of gasdermin pore assembly

**Fig. 2:**
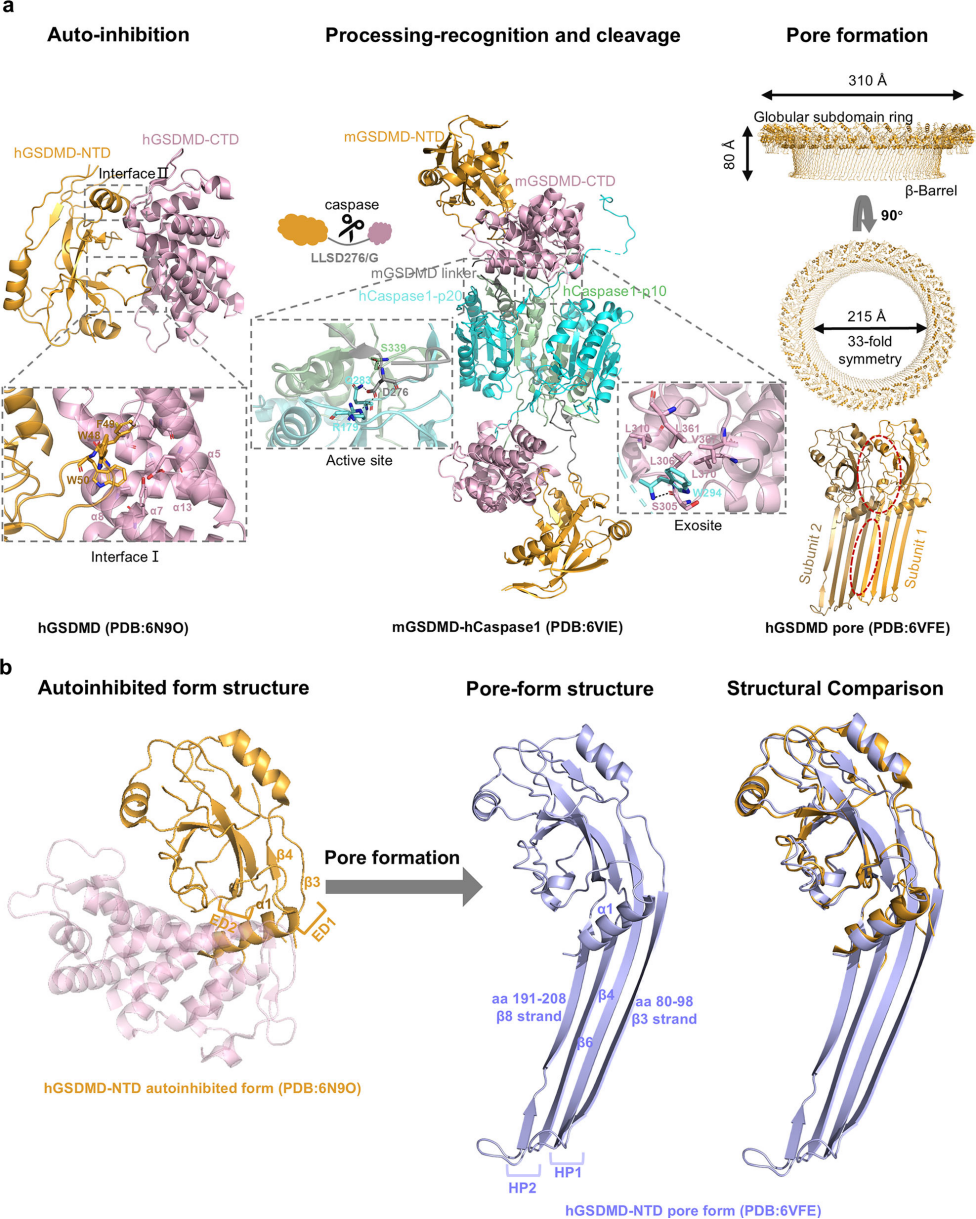
Step‑wise structural transitions of GSDMD. **a** Autoinhibited human GSDMD is unleashed when caspase‑1 engages the CTD exit and cleaves the inter‑domain linker; the free NTD oligomerizes into a 33‑subunit β‑barrel pore. **b** Conformational changes of hGSDMD-NTD before and after pore formation. The positively charged lipid-binding helix α1 is masked by hGSDMD-CTD in auto-inhibition (left), whereas in the pore-form helix α1 interacts with negatively charged lipids of the plasma membrane (middle). During pore formation, hGSDMD-NTD extension domains 1 and 2 (ED1 and ED2, in bright orange) in auto-inhibition, undergo a dramatic conformational change and become two transmembrane β hairpins (HP1 and HP2, in light blue).

Over the past years, high‑resolution structures have been reported for multiple GSDMs [13, 19, 74–77]. In well-established cleavage‑dependent activation model, gasdermins undergo a conserved sequence of structural transitions. They are synthesized as autoinhibited two‑domain proteins, in which the CTD suppresses the pore‑forming activity of the N‑terminal domain (NTD). Proteolytic cleavage releases the NTD, which binds to membranes enriched in acidic phospholipids and undergoes lateral oligomerization. During this process, each subunit re-organizes into two β‑hairpins that insert into the lipid bilayer; neighboring hairpins interweave to assemble a transmembrane β‑barrel. The expulsion of occluding lipids converts this “pre-pore” into an open conduit that induces pyroptotic cell death (Fig. 2).

In addition to driving lytic cell death, GSDM pores also play a crucial role in the unconventional secretion of the proinflammatory cytokines IL-1β and IL-18 [78]. The pore lumen is surrounded by four acidic patches (APs) that create a negatively charged electrostatic potential, which selectively facilitates the passage of basic cargoes while repelling acidic ones [74]. Although pro- and mature IL-1β and IL-18 are similar in size and much smaller than the GSDMD pore, proteolytic maturation increases their positive charge. This promotes precursor retention, selective secretion of the mature cytokines, and supports sustained inflammasome signaling. While several studies have shown that both GSDME and GSDMD can form pores in mitochondrial membranes, the structural organization and stoichiometry of these mitochondrial pores remain poorly defined [10, 11].

In an emerging cleavage-independent mechanism of human GSDMD activation [16], full-length GSDMD can be *S*-palmitoylated at Cys191, which loosen the interaction between the NT and CT domains, enabling the NT domain to form a 33-fold symmetric pore that is close to the GSDMD-NT pore but with prominent extra densities due to the retention of GSDMD-CT and the attached palmitate group. Moreover, autism-associated V41A mutation in GSDMD increases this palmitoylation modification that overcomes autoinhibition, leading to inflammasome- and cleavage-independent GSDMD activation [16]. This raises the intriguing question of whether cleavage-independent activation represents an evolutionarily conserved mechanism across a broader range of organisms.

**Fig. 3:**
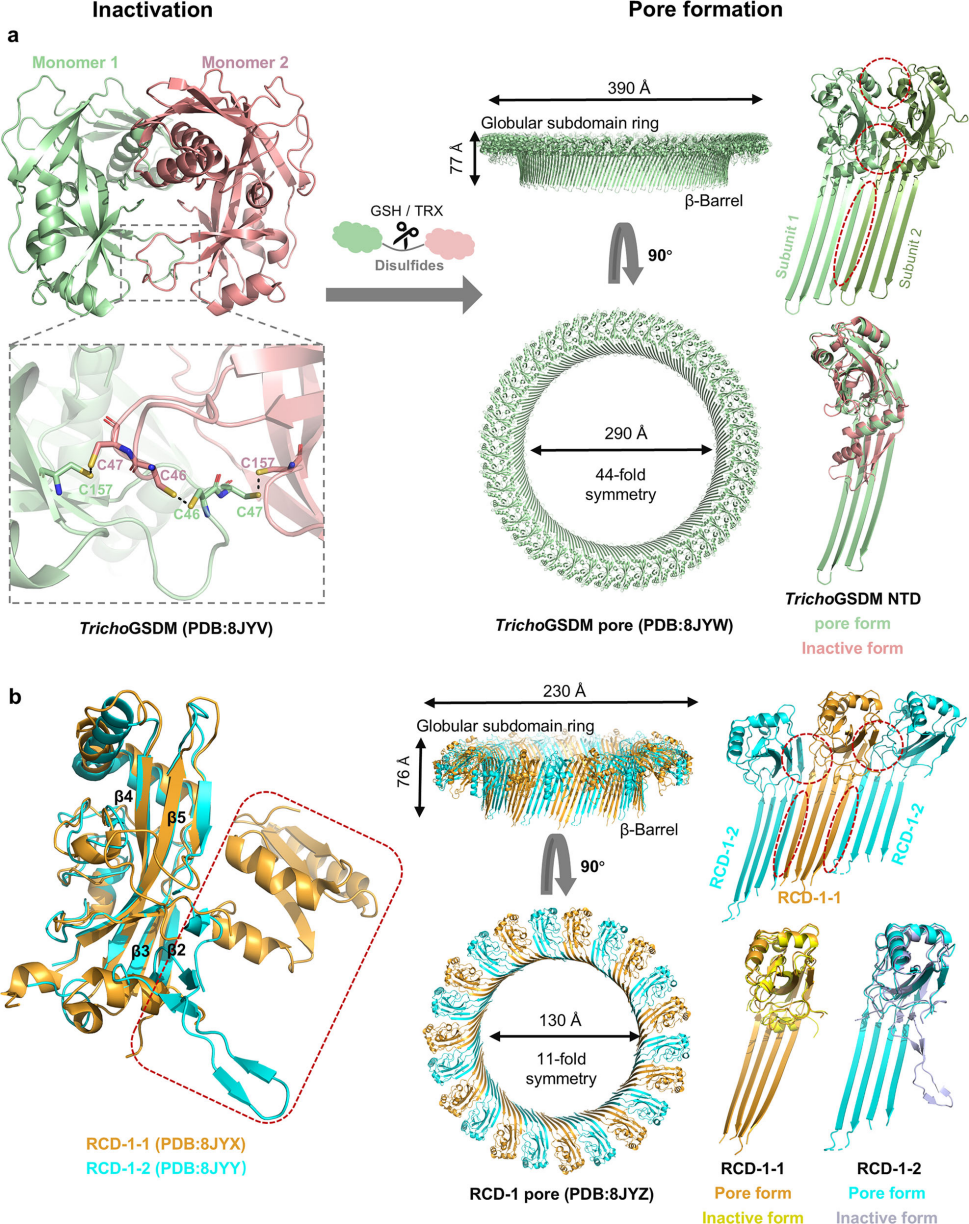
Cleavage-independent pore formation in basal GSDMs. **a** *Tricho*GSDM is activated through disulfide reduction by glutathione (GSH) or protein disulfide reductases, and assemble a giant 44‑mer pore. **b** *Neurospora* GSDM-like RCD‑1‑1 and RCD‑1‑2 are individually inert but heterodimerize at 1:1 to generate an 11‑subunit pore. Insets highlight disulfide or interface rearrangements that drive activation. The conformational changes of RCD-1-1 before (in yellow) and after (in bright orange) pore formation, RCD-1-2 before (in light blue) and after (in cyan) pore formation.

In the ancient metazoan *T. adhaerens*, a cleavage‑independent gasdermin (*Tricho*GSDM) is expressed that is homologous to human GSDME and PJVK [14]. *Tricho*GSDM consists only of the NTD and forms a disulfide‑linked homodimer (Fig. 1). Crystal structures reveal compactly folded dimers of *Tricho*GSDM: at both edges of the core β‑sheet, strands β4, β5, β8, and β9 assemble into an additional four‑stranded sheet that, together with helix α4, almost completely shields helix α1. In other gasdermins, this region is either disordered or forms a three‑stranded sheet that extends a short helix toward the C‑terminal domain. These structural features support an CTD‑independent autoinhibitory mechanism in which the compact folding and disulfide‑mediated dimerization restrain pore formation. During pore assembly, β4-β5 linker refolds into a β hairpin and become an extended hairpin (β4-β5). The loop between β7 and β8 folds into a long strand and new β8-β9 hairpin. The globular subdomains and the transmembrane β hairpins further stabilize inter‑protomer contacts that drive oligomerization, expansion and form the giant 44-fold symmetric pores (Fig. 3a). The protomer numbers and inner and outer diameters of *Tricho*GSDM pore is greater than any of mammalian GSDM pores, potentially enabling the release of larger or more abundant cytosolic contents.

Another cleavage-independent gasdermin is RCD-1 from *Neurospora crassa* [14]. The *rcd-1* alleles encode two nearly identical paralogues, RCD-1-1 and RCD-1-2, which share a common structural architecture characterized by a twisted central β-sheet (β1–β6) flanked by α-helices. However, their structures differ in flexible regions: RCD-1-1 contains an additional 10-residue loop that inserts orthogonally into the concave face of the C-terminal β-sheet, which is missing in RCD-1-2. Additionally, the loop that connects β2 to β3 and β4 to β5 of RCD‑1‑1 remains highly mobile, but it adopts an ordered conformation in RCD‑1‑2 (Fig. 3B). Under resting-state, both RCD-1-1 and RCD-1-2 show high membrane affinity due to high flexible loops and fully exposed lipid-binding sites. When co-present, RCD-1 and RCD-2 hetero‑oligomerize in an alternating fashion to form an 11-fold symmetric tiny pore. During assembly, the mobile loops connect β2 to β3 and β4 to β5 refold to form the transmembrane β hairpins that span the bilayer (Fig. 3b). The discovery of cleavage-independent gasdermin such as RCD‑1 highlights the mechanistic diversity in GSDM activation, and offers insight into the evolutionary pathways that shaped their activation strategies in mammalian.

## Gasdermins and neurodegenerative disease

**Fig. 4:**
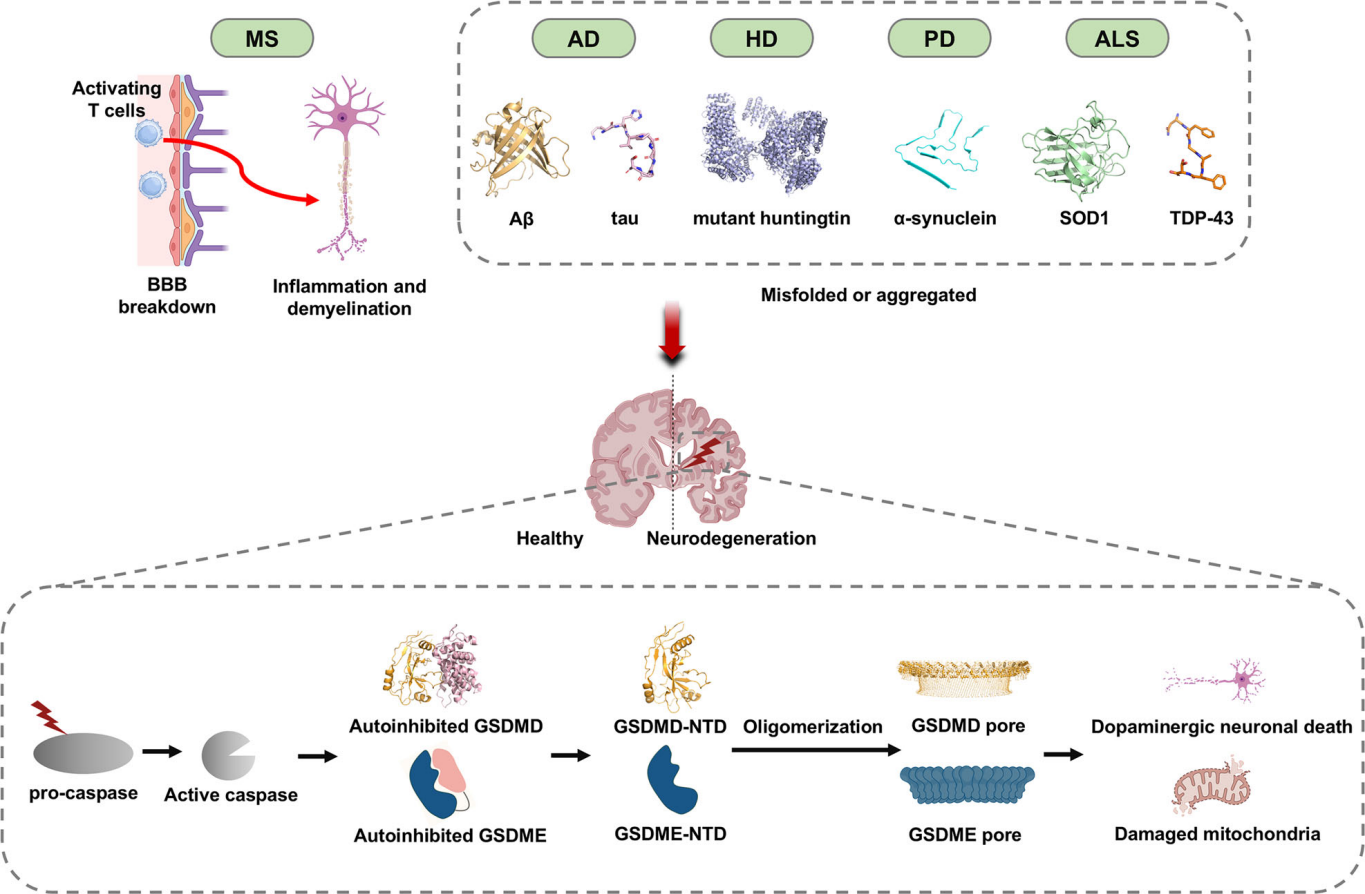
GSDMs in neurodegenerative diseases. Inflammasome‑ and caspase‑mediated cleavage of gasdermins is implicated in Alzheimer’s, Parkinson’s, Huntington’s, ALS and multiple sclerosis. The gasdermin pores permeabilize mitochondria or plasma membranes, intensify neuroinflammation, and accelerate neuronal loss, thereby feeding a vicious cycle of protein misfolding, immune activation and progressive degeneration. Created with BioRender.com.

Emerging data place the pore-forming proteins GSDMD and GSDME within the circuitry of chronic neuroinflammation that drives many neurodegenerative diseases [79]. Under physiological conditions, GSDMD is expressed in microglia, myeloid cells, and brain endothelial cells, while GSDME is present in neurons [11]. GSDMD transcription is induced by IRF2 and TLR4/NF-κB signaling, whereas GSDME expression is enhanced by the transcription factor Sp1 during pyroptosis [80, 81]. Persistent insults, such as misfolded proteins, mitochondrial toxins, or autoreactive lymphocytes, lead to inflammasome activation and release gasdermin N-terminal fragments that oligomerize within membranes. The resulting pores unleash IL-1β, IL-18, and cytochrome c, fueling a feed-forward loop in which innate immunity, adaptive immunity, and cell-intrinsic stress amplify one another, accelerating synapse loss, axon degeneration, and demyelination (Fig. 4).

### Age-related macular degeneration (AMD)

AMD is the leading cause of irreversible blindness in the elderly [82, 83]. The disease begins with drusen deposits, which are extracellular accumulations of lipids, proteins, nucleotides, and complement components that build up at the interface between retinal pigment epithelium (RPE) and Bruch’s membrane interface, followed by chronic RPE loss and secondary photoreceptor degeneration [84, 85]. Drusen components such as retrotransposon‑derived *Alu* RNA and Aβ fibrils activate NLRP3 and NLRC4 inflammasome in RPE death depend on GSDMD for execution [86–89]. Notably, RPE-restricted GSDMD can drive degeneration even in the absence of caspase-mediated cleavage, although the underlying mechanism remains unknown [90]. Additionally, phototoxicity and all-trans retinal (atRAL) stimulate GSDMD and GSDME activation, which is required for photoreceptor neurodegeneration [91, 92]. Mice deficient in *Gsdmd* or *Gsdme* are protected retina from neuroinflammation and photoreceptor loss, and exhibit preserved visual functions [92].

### Alzheimer’s disease (AD)

AD is the most common cause of dementia [93]. It begins with the presence of amyloid plaques and neurofibrillary tangles in the brain. During progress, amyloid‑β (Aβ) deposition and tau hyperphosphorylation define the histopathological lesions that trigger a broad inflammatory cascade [94]. Activated microglia and astrocytes release complement components and chemokines that recruit monocytes and T cells into the lesions, along with bursts of pro-inflammatory cytokines, ROS, and nitric oxide that damage synapses and vascular endothelium, leading to blood-brain barrier (BBB) leakage and maladaptive crosstalk between central and peripheral immune system [95]. In patients with AD, cleaved GSDMD is detectable in peripheral blood mononuclear cells and appears in the spleen before emerging in the brain, accompanied by caspase-1/8/11 activation [96, 97]. Within the CNS, cleaved GSDMD localizes to microglia, infiltrating macrophages and astrocytes clustered around Aβ plaques [98]. At the peak stage of Aβ deposition, GSDMD is activated in the brains of 5×FAD mice, with expression primarily in activated microglia (amoeboid-like), infiltrated macrophages, but not in resting microglia (ramified-like), astrocytes, neurons, or T cells [97]. Conditional deletion of Gsdmd using Cx3cr1-Cre in the 5×FAD mouse model reduces plaque burden and gliosis, preserves blood-brain-barrier integrity, limits early T cell expansion and alleviates neurocognitive impairments [97].

GSDME exerts a complementary, neuron-intrinsic effect. Its expression is elevated in the hippocampus of APP23/PS45 mice, and Aβ challenge of SH-SY5Y cells triggers caspase-3–dependent GSDME cleavage, driving pyroptotic cell death [99]. Silencing or deleting *Gsdme* rescues spatial memory and dampens microgliosis in APP23/PS45 animals [99]. Collectively, these findings place GSDMD and GSDME upstream drivers of both neurovascular dysfunction and maladaptive immune programming in AD.

### Amyotrophic lateral sclerosis (ALS)

ALS is a progressive neurodegenerative disorder characterized by selective loss of motor neurons and gradual motor decline [100]. Pathogenic protein species, including misfolded SOD1, aggregated TDP-43, and *C9orf72*-derived dipeptide repeat proteins, disrupt mitochondrial function and contribute to axon degeneration and neuron loss. In cortex and spinal cord tissues from ALS patients, GSDMD processing and IL-18 expression were upregulated in microglia [101]. Notably, the burden of cleaved GSDMD positive microglia in the precentral white matter inversely correlates with cortical neuron density, implicating the potential roles of GSDMD-mediated pyroptosis in neuron loss and ALS pathogenesis [102]. Similar pathologies were observed in TDP‑43^A315T^ mice, where microglial pyroptosis rises at symptom onset and correlates with neuronal degeneration [101]. In contrast to the microglia-associated GSDMD signal, GSDME is expressed in neurons and links mitochondrial damage to axon degeneration of ALS [11]. Mitochondrial toxins trigger caspase‑dependent GSDME cleavage and rapid N‑terminal translocation to axon mitochondria, resulting in mitochondrial membrane depolarization, axon transport defects, and synapse retraction. Aggregated ALS-linked TDP-43, as well as poly (PR) (a *C9orf72* repeat–associated dipeptide), induce NLRP3 inflammasome in cultured microglia, amplifying motor neuron toxicity, and can also engage GSDME-mediated mitochondrial damage and neurite loss [11].

Functionally, *Gsdme* knockdown in ALS patient iPSC-derived motor neurons preserves synapses, and *Gsdme* knockou**t** in SOD1^G93A^ mice extends survival, improves motor performance, protects motor neurons, and dampens neuroinflammation and disease severity [11]. However, in SOD1^G93A^ mice, although cleaved GSDMD is detectable in spinal-cord microglia, *Gsdmd* deletion yields minimal benefit and may even shorten survival, underscoring isoform- and cell-type–specific roles [103]. Collectively, these data argue that therapeutic strategies for ALS will likely need to prioritize neuronal GSDME, or implement coordinated, cell-type tailored modulation of both GSDME and GSDMD, rather than rely on blanket inflammasome blockade.

### Huntington’s disease (HD)

Huntington’s disease is an autosomal dominantly inherited neurodegenerative disease caused by abnormal expansion of CAG trinucleotide repeats in the *Huntingtin* (HTT) gene [104]. This mutation leads to the production of mutant huntingtin protein (mHTT), which aggregates and forms inclusion bodies within neurons. Although no studies have directly investigated the role of GSDMs in HD, patients with HD have marked neuroinflammation features at the site of the brain lesion [105]. One study reported elevated expression of NLRP3 in the PBMC of HD patients, along with significantly increased levels of IL-1β in the plasma, striatum, and cerebral cortex [106, 107]. In transgenic R6/2 HD model mice, both NLRP3 and active caspase-1 levels were higher in the striatum compared to wild-type controls [108]. Notably, treatment with the pan-caspase inhibitor zVAD-fmk delayed disease progression and extended survival in this model [109]. These findings suggest that NLRP3 inflammasome activation and subsequent pyroptosis may play a critical role in HD pathogenesis. However, whether GSDMs directly mediate pyroptotic neuronal death in HD remains unknown.

### Multiple sclerosis (MS)

Multiple sclerosis is a prototypical neuroinflammatory disorder driven by autoimmune responses that damages the myelin sheath and underlying axon [110]. CNS tissues from patients with progressive MS shows chronic active demyelinating lesions with significantly elevated expression of GSDMD, NINJ1, IL-1β, and IL-18 [111]. Similarly, in the cuprizone model, *Gsdmd* expression markedly upregulated in CNS macrophages and oligodendrocytes within the central corpus callosum (CCC) region. Mice with *Gsdmd* deletion shown reduced gliosis, demyelination and axon loss after cuprizone exposure. Notably, GSDMD deletion does not alter mature Olig2+ oligodendrocyte numbers, but enhance PDGFRα+ oligodendrocyte precursor cell proliferation and remyelination [111]. In experimental autoimmune encephalomyelitis (EAE) model, GSDMD in peripheral myeloid cells, but not in microglia, drives pro-inflammatory cytokines production, that promote expansion and differentiation of reactive T cells in secondary lymphoid organs [112]. *Gsdmd* knockout dampens T‑cell priming, limits T‑cell infiltration into the CNS, and ameliorates neuroinflammation and demyelination, indicating that GSDMD is required for full EAE pathogenesis [112].

Additionally, recent data show increased *Gsdme* expression in PBMCs and brain lesions of MS patients [113]. In human microglial HMC3 cells, GSDME disturbs microglia-mediated myelin debris degradation via autophagy. In the EAE model, caspase-3/GSDME activation triggers microglia pyroptosis critically mediated the progression of neuroinflammation and white matter demyelination. In vivo, systemic *Gsdme* deficiency markedly attenuates neuroinflammation and white‑matter demyelination in EAE [113]*. Gsdme* has been reported as the predominant gasdermin transcript detected in many neuronal populations, raising the possibility of neuron‑intrinsic roles in MS and related demyelinating conditions [11]. Whether GSDME directly drives neuronal degeneration or acts mainly through microglia‑mediated mechanisms remains unresolved. Targeted, cell type specific loss‑of‑function studies will be needed to separate these possibilities.

### Parkinson’s disease (PD)

Progressive loss of dopaminergic neurons in the substantia nigra pars compacta (SNc), and the accumulation of Lewy bodies are hallmark features of PD. Misfolded α-syn fibrils, the main component of Lewy bodies, potently activate the NLRP3 inflammasome in microglia. In the MPTP (1-methyl-4-phenylpyridinium) neurotoxin model of PD, transcripts of pyroptosis-associated genes, including *Nlrp3*, *Casp1* and *Gsdmd* were upregulated after MPTP exposure [114]. Genetic deletion of *Gsdmd* reduces neuroinflammation and neuropathology in both MPTP- and α-synuclein^A53T^ overexpression-induced PD mouse models [115, 116]. Furthermore, microglia-specific conditional *Gsdmd* knockout attenuated MPTP-induced neuroinflammation, dopaminergic neuron loss, and motor dysfunction in vivo, underscoring the central role of microglial pyroptosis in disease progression [117].

## Therapeutic modulation of gasdermin

Therapeutic efforts have so far focused on GSDMD and center on small-molecule inhibitors [118] (Table 1). Compounds such as disulfiram (and its copper adduct CuET), necrosulfonamide (NSA), dimethyl fumarate (DMF) and LDC7559 all share a common mechanism: covalent modification of the nucleophilic cysteine residue (Cys191 in human GSDMD, Cys192 in mouse) to prevent oligomerization and pore formation. These agents have other molecular targets, and their broad thiol reactivity also affects caspases, MLKL and other redox‑sensitive proteins, limiting their on‑target selectivity and durability. Drug development for other GSDM members is still in its infancy. CuET has been shown to cross-link GSDME in addition to GSDMD; however, no truly selective small‑molecule inhibitor of GSDME has been reported [119]. Beyond small molecules, biologics such as nanobodies are gaining traction. Several camelid single-domain antibodies targeting GSDMD and GSDME have been developed through phage display and exhibit nanomolar affinity, offering highly selective, non-covalent inhibition that avoids many of the liabilities associated with covalent small molecules [120].

**Table 1:** Overview of potent inhibitors targeting GSDMD.

Name	Chemical structure	Potency (Method)	Selectivity	Refs.	Mechanisms of action
Necrosulfonamide		*K*_d_: 32 µM (SPR)	Binding Cys86 on MLKL	[104, 105]	Targeting Cys191 on GSDMD and inhibiting GSDMD pore formation
LDC7559		Not available	Inhibiting PMA-induced NET formation	[39, 109]	Binding to GSDMD and inhibiting inflammasome activation
Dimethyl fumarate		Not available	Inhibiting GSDME at Cys45	[45, 112]	Targeting Cys191 on GSDMD and inhibiting GSDMD pore formation
Disulfiram		IC_50_: 0.26 µM (MST)	No inhibiting GSDMA3, but binding caspase-1 and caspase-11	[115]	Targeting Cys191 on GSDMD and inhibiting GSDMD pore formation
Itaconate		Not available	Inhibiting SDH activity and triggering the NRF2 and ATF3 transcription factors	[77, 121]	Targeting Cys77 on GSDMD and affecting caspase-1/GSDMD interplay

*K*_d_: dissociation constant; IC_50_: half-maximum inhibitory concentration.

*SPR* surface plasmon resonance, *MST* microscale thermophoresis.

### Cysteine-targeting compounds

*Necrosulfonamide (NSA)* was the first GSDMD inhibitor initially identified as an MLKL inhibitor that binds to Cys86 and blocks necroptosis [121]. Later studies revealed NSA also covalently modifies GSDMD at Cys191 (Cys192 in mouse), preventing oligomerization and N-terminal pore formation, with a dissociation constant (*K*_d_) of ~32 μM [122]. However, NSA exhibits off-target effects, suppressing LPS-induced transcription and caspase-1 activation upstream of pyroptosis. Despite this, NSA has shown efficacy in reducing inflammatory cytokine secretion and improving survival in murine models of sepsis [122]. It also attenuates microglial activation, reactive astrogliosis, and MPTP-induced cognitive impairments in Parkinson’s disease (PD) models [123]. In the mouse model of spinal cord injury (SCI), NSA ameliorates mitochondrial dysfunction and neuronal death. Intraperitoneal administration of NSA improves the motor function and spinal edema of SCI mice [124]. In a rat model of AD, NSA alleviated the abnormally high hippocampal expression proinflammatory cytokines, β-amyloid and phosphorylated tau protein with amending spatial learning and memory deficits in Morris water and Y-mazes tests [125].

*LDC7559*, a pyrazolo-oxazepine compound, was originally identified as an inhibitor of neutrophil extracellular trap (NET) formation. It does not affect NADPH oxidase, MPO activity, or phagocytosis, but inhibits PMA-induced NETs with an IC₅₀ of 5.61 μM [42]. Although its exact mechanism is unclear, LDC7559 directly binds the GSDMD N-terminal domain, rather than interfering with its cleavage [42]. Later studies attributed its anti-pyroptotic effects to off-target activity, particularly PFKL agonism and metabolic reprogramming [126]. In the rat model of subarachnoid hemorrhage, LDC7559 treatment reduced microglial activation, neuronal pyroptosis and neuroinflammation. Mice that received LDC7559 had improved neurological deficits, motor function and histological brain edema after brain injury [127]. In mouse model of traumatic brain injury (TBI), LDC suppressed cytokines release, microglia activation and improved neural recovery [128].

*Dimethyl fumarate (DMF)* initially approved as a first-line oral treatment for relapsing forms of multiple sclerosis (MS), has been repurposed as a potent GSDMD inhibitor, with an IC₅₀ of 0.26 μM [57]. DMF promotes GSDMD succinate through reacting with Cys[191], Cys [57] and Cys [77] to form S-(2-succinyl)-cysteines. These modifications of GSDMD reduces its binding to caspase 1, blocking its processing, oligomerization, and pore formation. In addition, DMF succinylates the cysteine residue (Cys [45]) of GSDME, preventing its processing, oligomerization, and pyroptosis [57]. In the EAE model, DMF reduces T cell infiltration, delays disease onset, and alleviates neuropathology and demyelination.

DMF is also a known activator of the Nrf2 pathway. In isolated hippocampal neurons, DMF, but not its metabolite monomethyl fumarate, enhances Nrf2 abundance and nuclear translocation. In models of AD and PD, DMF significantly upregulates the Nrf-2 pathway, mitigates dopaminergic neuronal degeneration, and improves behavioral outcomes [129]. It also reduces neuroinflammation and improves cognitive function, but without altering amyloid-β clearance in AD [130].

*Disulfiram (DSF)*, an FDA-approved aldehyde-dehydrogenase inhibitor, was discovered in a high-throughput screen as a covalent blocker of GSDMD [131]. At nanomolar concentrations, DSF or its metabolite diethyldithiocarbamate (DDC) covalently modifies Cys191 in GSDMD, blocking oligomerization without affecting caspase-mediated cleavage, preventing IL-1β/IL-18 releases and pyroptotic cell death. Complexation with Cu2+ yields the bis-diethyldithiocarbamate copper adduct (CuET), which boosts potency ~20-fold and cross-links reactive cysteines in GSDME [132].

DSF shows broad therapeutic potential, providing protection against sepsis, colitis, TBI, and lupus by suppressing pyroptosis [133, 134]. However, like other Cys191-targeting agents, DSF has notable off-target effects. Subsequent studies revealed that GSDMD is not the primary target of disulfiram; rather, DSF selectively inhibits the NLRP3 inflammasome, without effecting AIM2 or NLRC4 inflammasomes, by directly modulating NLRP3 palmitoylation at Cys126 [55]. Despite these limitations, DSF’s well-established clinical use, oral bioavailability, and copper-enhanced activity make it an attractive scaffold for further pharmacological optimization.

*Itaconate* is a unique regulatory metabolite induced upon Toll-like receptor (TLR) stimulation in myeloid cell [135]. It plays a central role in metabolic reprogramming during immune response, particularly in macrophages [136]. Recent studies suggest that intracellular itaconate accumulation promotes itaconate-based PTM of GSDMD at Cys77, preventing its processing and pore formation. GSDMD itaconation confers the cell tolerance to pyroptotic cell death and tissue damage during inflammation [136]. Beyond GSDMD, endogenous itaconate also modifies cysteine 13 of IRAK4, disrupting its autophosphorylation and activation, promoting degradation of nuclear factor (NF)-κB, and modulating global ubiquitination patterns [137]. Dimethyl itaconate (DI), a membrane-permeable derivative of itaconate, significantly reduces brain Aβ deposition, decreases hippocampal and cortical neuron loss in the APP/PS1 transgenic mouse model of AD [138]. In addition, itaconate alleviates anesthesia/surgery-induced cognitive impairment by activating Nrf2-dependent anti-inflammatory and neurogenic pathways [139].

DI has also been shown to reduce BBB disruption, suppress MMP3/MMP9 production, inhibit microglia activation, and ameliorate disease severity in the chronic EAE model. Furthermore, supplementation with 4-octyl itaconate (OI) restores oxidative metabolism of glucose, glutamine, and fatty acids, attenuates pro-inflammatory activation and neurodegeneration, and improves long-term neurological outcomes in TBI [140]. Conversely, in tissue-resident alveolar macrophages, itaconate enhances cytokine production and NLRP3 inflammasome activation and exacerbates acute lung injury through mechanisms independent of Nrf2- and GSDMD signaling, underscoring its context-dependent effects [141].

### Emerging paradigms in gasdermin inhibition

*Oligomerization blockers:* Most legacy GSDMD inhibitors act by covalently modifying the conserved Cys191/Cys192 residue; however, this thiol-based strategy risks broad off-target reactivity. In a recent study, Hu et al. adopted an alternative approach by screening for compounds that bind oligomerization interface I and identified two repurposed, orally available drugs: metatinib anhydrous (MET/VEGFR tyrosine‑kinase inhibitor) and cefcapene pivoxil HCl (antibiotic) [142]. These compounds suppress GSDMD-driven pyroptosis without perturbing caspase-1/4/11 activation or targeting the Cys191/192 hotspot. Instead, they form hydrogen and halogen bonds with Cys56, Asn58 and Glu143 on interface I, sterically blocking head-to-tail oligomerization. In primary macrophages, both drugs dose-dependently reduce LDH release and IL-1β/IL-18 secretion, and significantly alleviate sepsis in mice models [142]. Although these compounds have not yet been evaluated in models of neurodegeneration, their oral bioavailability and clinically de-risked status make them promising starting points for medicinal chemistry optimization toward selective, non-covalent inhibition of GSDM pore formation.

*Nanobody neutralizers:* A growing repertoire of camelid single-domain antibodies (VHHs) has been developed to target GSDMD and GSDME pore formation. A panel of six VHHs raised against human GSDMD was recently developed [120, 143]. Crystal structures of VHH–GSDMD complexes revealed that CDR3 loops of the nanobodies bind across the β7–β8 rim of the N-terminal domain, sterically blocking pore assembly without interfering with caspase-mediated cleavage. Both cytosolic expression and extracellular delivery of these VHHs inhibited pore formation, pyroptotic lysis, and IL‑1β release, highlighting their potential as precise anti‑GSDM tools [143]. In addition, isPLA-seq screening against GSDME identified single‑domain antibodies that recognize the GSDME-NT domain [144]. These nanobodies blocks caspase-3-induced GSDME pore formation, reduces cisplatin‑triggered cytokine release, and mitigates chemotherapeutic lung injury in mice. Furthermore, systemic administration of GSDMD-targeting nanobodies effectively prevent BBB disruption caused in models of LPS and *K. pneumoniae* infection [145]. Nanobodies represent a novel, non-covalent, and epitope-specific approach to inhibit GSDM pore formation while preserving upstream signaling. Their modular structure supports engineering for extended half-life, intracellular delivery, or bispecific targeting, making them as a promising platform for selectively neutralizing GSDM activity in neuroinflammation and neurodegenerative diseases.

*Cleavage-site peptidomimetics:* Inflammatory caspases recognize the FLTD cleavage motif in GSDMD [146]. Recently, Yang et al. engineered Ac-FLTD-CMK (N-acetyl-Phe-Leu-Thr-Asp-chloromethyl ketone), an irreversible active-site inhibitor that docks in the S4–S1 pockets of caspases-1, -4, -5, and murine caspase-11, forming a covalent bond with the catalytic cysteine and block GSDMD processing [147]. In primary BMDM, Ac-FLTD-CMK effectively prevents GSDMD cleavage downstream of both canonical (NLRP3) and non-canonical (caspase-11) inflammasomes, abolishing LDH release, and IL-1β/IL-18 secretion without affecting caspase-3 or apoptotic signaling. In a mice model of TBI, intraventricular injection of Ac-FLTD-CMK significantly reduces neuroinflammation, cytokines levels and attenuates neuron death, BBB disruption and brain edema after brain injury [148]. Although its cell permeability and metabolic stability remain to be fully characterized, this proof-of-concept study establishes a peptidomimetic approach to inhibit GSDM activation by targeting the upstream protease switch rather than the pore-forming mechanism itself.

Notably, each of these strategies has limitations. Oligomerization blockers require further optimization to improve potency and isoform selectivity, and their efficacy in neurodegeneration models remains untested. Nanobody neutralizers, while highly specific, face challenges with systemic delivery, BBB penetration, and potential immunogenicity. Cleavage-site peptidomimetics are limited by poor cell permeability, rapid metabolic degradation. Additionally, instead of blocking gasdermin protein processing or pore assembly, approaches that modulate gasdermin expression, either through antisense oligonucleotides (ASOs) or small interfering RNAs (siRNAs) at the mRNA level, or proteolysis-targeting chimeras (PROTACs) at the protein level, could offer highly selective interventions, but require further investigation.

## Conclusions and future perspectives

A decade after GSDMs were identified as the executioner of inflammatory cell death, structural and mechanistic studies have revealed both a unifying framework and striking diversity in gasdermin activation. Two broad modes of activation have emerged. The canonical route involves proteolytic cleavage: severing the interdomain linker disengages the C-terminal “brake,” releasing the N-terminal pore-forming module. In contrast, loss of autoinhibition via pathogenic mutations (like V41A in human GSDMD), alternative splicing, post-translational modifications, or pathogen-encoded effectors can also license the pyroptotic pore formation independent of enzymic proteolysis. In both scenarios, the free N-terminal domain binds to membranes, oligomerizes, and perforates the bilayer. However, the kinetics, subcellular localization, and functional consequences of pore formation vary significantly by cell type, stimulus, and gasdermin isoforms. Mapping these kinetic and spatial control points is now a central challenge. Key open questions include: What checkpoints govern pore growth after initial membrane binding? How do lipids, post-translational modifications, and interacting proteins bias outcomes ranging from sublytic cytokine release to full lytic cell death?

These questions are especially pressing in the nervous system. Research on GSDMs in neurodegenerative diseases remains limited, and evidence of gasdermin activation in human brain tissue is restricted to a few small postmortem studies with limited sample sizes and regional coverage. Most available data are cross-sectional and derived primarily from rodent models, which may not fully capture human disease processes. Moreover, microglia, astrocytes, neurons, oligodendrocytes, and infiltrating immune cells express distinct gasdermin repertoires, yet a cell-resolved atlas linking isoform, trigger, and outcome across neurodegenerative conditions remains lacking. For instance, does GSDME-driven pyroptosis in oligodendrocytes accelerate demyelination? Could sublytic GSDMB activity in astrocytes serve as a controlled cytokine release valve? Progress will depend on refined tools: isoform-specific reporters, conditional knock-ins from modeling patient variants, spatial proteomics, and in situ structural probes that move beyond conventional measurements of IL-1β, LDH measurements and propidium iodide (PI) uptake assay.

Therapeutically, gasdermins have evolved from being viewed as “undruggable” pores to tractable molecular switches. First-generation Cys191/192 alkylators demonstrated druggability but are limited by promiscuous thiol reactivity. Next-wave approaches are already emerging: noncovalent interface-I “wedges”, nanobody “caps,” peptidyl caspase “traps” that block activating cleavage, and metabolite-derived allosteric modulators. A particularly promising direction is modulation rather than outright inhibition, such like tuning pore diameter, dwell time, or membrane distribution to favor cytokine release without triggering catastrophic lysis. Achieving such control will likely require precise manipulation of gasdermin post-translational modifications (e.g., palmitoylation, phosphorylation, itaconation, ADP-ribosylation) and lipid microenvironments that regulate pore expansion.

Overall, the gasdermin field has progressed from defining how pore-forming mechanisms to confronting the harder question of when, where, and to what effect those pores form in vivo. Translational progress will require a multidisciplinary approach, leveraging high‑resolution structural data for drug design, systemic-level omics to map context-specific activation circuits, and disease models that capture both the acute blaze of sepsis and the slow burn of chronic neuroinflammation [149]. Bringing together insights across these scales will be essential for developing precise GSDM modulators that ideally suppress pathological inflammation while preserving the antimicrobial or antitumor functions inherent to physiological GSDM pore formation.

## References

[1] Fang Y, Tian S, Pan Y, Li W, Wang Q, Tang Y, et al. Pyroptosis: a new frontier in cancer. Biomed Pharmacother. 2020;121:109595. 31710896 10.1016/j.biopha.2019.109595

[2] Bai Y, Pan YD, Liu X. Mechanistic insights into gasdermin-mediated pyroptosis. Nat Rev Mol Cell Biol. 2025;26:501–521. 40128620 10.1038/s41580-025-00837-0

[3] Yu P, Zhang X, Liu N, Tang L, Peng C, Chen X. Pyroptosis: mechanisms and diseases. Signal Transduct Target Ther. 2021;6:128. 33776057 10.1038/s41392-021-00507-5 PMC8005494

[4] Gong Y, Fan Z, Luo G, Yang C, Huang Q, Fan K, et al. The role of necroptosis in cancer biology and therapy. Mol Cancer. 2019;18:100. 31122251 10.1186/s12943-019-1029-8 PMC6532150

[5] Dai Z, Liu WC, Chen XY, Wang X, Li JL, Zhang X. Gasdermin D-mediated pyroptosis: mechanisms, diseases, and inhibitors. Front Immunol. 2023;14:1178662. 37275856 10.3389/fimmu.2023.1178662 PMC10232970

[6] He WT, Wan HQ, Hu LC, Chen PD, Wang X, Huang Z, et al. Gasdermin D is an executor of pyroptosis and required for interleukin-1β secretion. Cell Res. 2015;25:1285–98. 26611636 10.1038/cr.2015.139 PMC4670995

[7] Angosto-Bazarra D, Alarcon-Vila C, Hurtado-Navarro L, Banos MC, Rivers-Auty J, Pelegrin P. Evolutionary analyses of the gasdermin family suggest conserved roles in infection response despite loss of pore-forming functionality. BMC Biol. 2022;20:9. 34996441 10.1186/s12915-021-01220-z PMC8742441

[8] Cookson BT, Brennan MA. Pro-inflammatory programmed cell death. Trends Microbiol. 2001;9:113–4. 11303500 10.1016/s0966-842x(00)01936-3

[9] Miao N, Wang Z, Wang Q, Xie H, Yang N, Wang Y, et al. Oxidized mitochondrial DNA induces gasdermin D oligomerization in systemic lupus erythematosus. Nat Commun. 2023;14:872. 36797275 10.1038/s41467-023-36522-z PMC9935630

[10] Miao R, Jiang C, Chang WY, Zhang HW, An JS, Ho F, et al. Gasdermin D permeabilization of mitochondrial inner and outer membranes accelerates and enhances pyroptosis. Immunity. 2023;56:2523–2541. 37924812 10.1016/j.immuni.2023.10.004 PMC10872579

[11] Neel DV, Basu H, Gunner G, Bergstresser MD, Giadone RM, Chung H, et al. Gasdermin-E mediates mitochondrial damage in axons and neurodegeneration. Neuron. 2023;111:1222–40 e1229. 36917977 10.1016/j.neuron.2023.02.019 PMC10121894

[12] Rogers C, Erkes DA, Nardone A, Aplin AE, Fernandes-Alnemri T, Alnemri ES. Gasdermin pores permeabilize mitochondria to augment caspase-3 activation during apoptosis and inflammasome activation. Nat Commun. 2019;10:1689. 30976076 10.1038/s41467-019-09397-2 PMC6459836

[13] Johnson AG, Mayer ML, Schaefer SL, McNamara-Bordewick NK, Hummer G, Kranzusch PJ. Structure and assembly of a bacterial gasdermin pore. Nature. 2024;628:657–63. 38509367 10.1038/s41586-024-07216-3 PMC11771145

[14] Li Y, Hou Y, Sun Q, Zeng H, Meng F, Tian X, et al. Cleavage-independent activation of ancient eukaryotic gasdermins and structural mechanisms. Science. 2024;384:adm9190. 38662913 10.1126/science.adm9190

[15] Zhou B, Jiang ZH, Dai MR, Ai YL, Xiao L, Zhong CQ, et al. Full-length GSDME mediates pyroptosis independent from cleavage. Nat Cell Biol. 2024;26:1545–57. 38997456 10.1038/s41556-024-01463-2

[16] Du G, Healy LB, David L, Walker C, El-Baba TJ, Lutomski CA, et al. ROS-dependent S-palmitoylation activates cleaved and intact gasdermin D. Nature. 2024;630:437–46. 38599239 10.1038/s41586-024-07373-5 PMC11283288

[17] Angosto-Bazarra D, Guijarro A, Pelegrin P. Evolution of the gasdermin family and pyroptosis. Dev Comp Immunol. 2023;149:105060. 37734430 10.1016/j.dci.2023.105060

[18] Saeki N, Usui T, Aoyagi K, Kim DH, Sato M, Mabuchi T, et al. Distinctive expression and function of four GSDM family genes (GSDMA-D) in normal and malignant upper gastrointestinal epithelium. Genes Chromosomes Cancer. 2009;48:261–71. 19051310 10.1002/gcc.20636

[19] Ruan JB, Xia SY, Liu X, Lieberman J, Wu H. Cryo-EM structure of the gasdermin A3 membrane pore. Nature. 2018;557:62. 29695864 10.1038/s41586-018-0058-6 PMC6007975

[20] Kumar S, Rathkolb B, Budde BS, Nürnberg P, de Angelis MH, Aigner B, et al. Gsdma is a novel ENU-induced mutant mouse line for studying the function of Gasdermin A3 in the hair follicle and epidermis. J Dermatol Sci. 2012;67:190–2. 22682752 10.1016/j.jdermsci.2012.05.001

[21] Li J, Zhou Y, Yang T, Wang N, Lian XH, Yang L. Gsdma3 is required for hair follicle differentiation in mice. Biochem Biophys Res Commun. 2010;403:18–23. 20977888 10.1016/j.bbrc.2010.10.094

[22] LaRock DL, Johnson AF, Wilde S, Sands JS, Monteiro MP, LaRock CN. Group A Streptococcus induces GSDMA-dependent pyroptosis in keratinocytes. Nature. 2022;605:527–31. 35545676 10.1038/s41586-022-04717-x PMC9186297

[23] Deng W, Bai Y, Deng F, Pan Y, Mei S, Zheng Z, et al. Streptococcal pyrogenic exotoxin B cleaves GSDMA and triggers pyroptosis. Nature. 2022;602:496–502. 35110732 10.1038/s41586-021-04384-4 PMC9703647

[24] Billman ZP, Kovacs SB, Wei B, Kang K, Cisse OH, Miao EA. Caspase-1 activates gasdermin A in non-mammals. eLife. 2024;12:RP92362 38497531 10.7554/eLife.92362 PMC10948149

[25] Kang MJ, Yu HS, Seo JH, Kim HY, Jung YH, Kim YJ, et al. GSDMB/ORMDL3 variants contribute to asthma susceptibility and eosinophil-mediated bronchial hyperresponsiveness. Hum Immunol. 2012;73:954–9. 22732088 10.1016/j.humimm.2012.06.009

[26] Zhong X, Zeng H, Zhou Z, Su Y, Cheng H, Hou Y, et al. Structural mechanisms for regulation of GSDMB pore-forming activity. Nature. 2023;616:598–605. 36991125 10.1038/s41586-023-05872-5

[27] Zhou Z, He H, Wang K, Shi X, Wang Y, Su Y, et al. Granzyme A from cytotoxic lymphocytes cleaves GSDMB to trigger pyroptosis in target cells. Science. 2020;368:eaaz7548 32299851 10.1126/science.aaz7548

[28] Yin H, Zheng J, He Q, Zhang X, Li X, Ma Y, et al. Insights into the GSDMB-mediated cellular lysis and its targeting by IpaH7.8. Nat Commun. 2023;14:61. 36599845 10.1038/s41467-022-35725-0 PMC9813358

[29] Wang C, Shivcharan S, Tian T, Wright S, Ma D, Chang J, et al. Structural basis for GSDMB pore formation and its targeting by IpaH7.8. Nature. 2023;616:590–7. 36991122 10.1038/s41586-023-05832-z PMC10115629

[30] Rana N, Privitera G, Kondolf HC, Bulek K, Lechuga S, De Salvo C, et al. GSDMB is increased in IBD and regulates epithelial restitution/repair independent of pyroptosis. Cell. 2022;185:283–98 e217. 35021065 10.1016/j.cell.2021.12.024 PMC8879997

[31] Li X, Christenson SA, Modena B, Li H, Busse WW, Castro M, et al. Genetic analyses identify GSDMB associated with asthma severity, exacerbations, and antiviral pathways. J Allergy Clin Immunol. 2021;147:894–909. 32795586 10.1016/j.jaci.2020.07.030 PMC7876167

[32] Saleh NM, Raj SM, Smyth DJ, Wallace C, Howson JM, Bell L, et al. Genetic association analyses of atopic illness and proinflammatory cytokine genes with type 1 diabetes. Diabetes Metab Res Rev. 2011;27:838–43. 22069270 10.1002/dmrr.1259 PMC3816329

[33] Tamura M, Tanaka S, Fujii T, Aoki A, Komiyama H, Ezawa K, et al. Members of a novel gene family, Gsdm, are expressed exclusively in the epithelium of the skin and gastrointestinal tract in a highly tissue-specific manner. Genomics. 2007;89:618–29. 17350798 10.1016/j.ygeno.2007.01.003

[34] Watabe K, Ito A, Asada H, Endo Y, Kobayashi T, Nakamoto K, et al. Structure, expression and chromosome mapping of MLZE, a novel gene which is preferentially expressed in metastatic melanoma cells. Jpn J Cancer Res. 2001;92:140–51. 11223543 10.1111/j.1349-7006.2001.tb01076.x PMC5926699

[35] Wei J, Xu Z, Chen X, Wang X, Zeng S, Qian L, et al. Overexpression of GSDMC is a prognostic factor for predicting a poor outcome in lung adenocarcinoma. Mol Med Rep. 2020;21:360–70. 31939622 10.3892/mmr.2019.10837 PMC6896373

[36] Wang S, Chang CW, Huang J, Zeng S, Zhang X, Hung MC, et al. Gasdermin C sensitizes tumor cells to PARP inhibitor therapy in cancer models. J Clin Invest. 2024;134:e166841 37883181 10.1172/JCI166841 PMC10760963

[37] Zhang JY, Zhou B, Sun RY, Ai YL, Cheng K, Li FN, et al. The metabolite α-KG induces GSDMC-dependent pyroptosis through death receptor 6-activated caspase-8. Cell Res. 2021;31:980–97. 34012073 10.1038/s41422-021-00506-9 PMC8410789

[38] Hou J, Zhao R, Xia W, Chang CW, You Y, Hsu JM, et al. PD-L1-mediated gasdermin C expression switches apoptosis to pyroptosis in cancer cells and facilitates tumour necrosis. Nat Cell Biol. 2020;22:1264–75. 32929201 10.1038/s41556-020-0575-z PMC7653546

[39] Pandey SP, Yang D, Hedden L, Laughlin CR, Wang W, Soto AS, et al. Gasdermin C cleavage by Cathepsin S modulates Rab7 vesicles in intestinal epithelial cells to amplify anti-helminth immunity. Immunity. 2025;58:2439–2455.e8. 40701157 10.1016/j.immuni.2025.06.018 PMC12380176

[40] Kanneganti A, Malireddi RKS, Saavedra PHV, Walle LV, Van Gorp H, Kambara H, et al. GSDMD is critical for autoinflammatory pathology in a mouse model of Familial Mediterranean Fever. J Exp Med. 2018;215:1519–29. 29793924 10.1084/jem.20172060 PMC5987922

[41] Liu Z, Wang C, Yang J, Chen Y, Zhou B, Abbott DW, et al. Caspase-1 engages full-length gasdermin D through two distinct interfaces that mediate caspase recruitment and substrate cleavage. Immunity. 2020;53:106–14 e105. 32553275 10.1016/j.immuni.2020.06.007 PMC7382298

[42] Sollberger G, Choidas A, Burn GL, Habenberger P, Di Lucrezia R, Kordes S, et al. Gasdermin D plays a vital role in the generation of neutrophil extracellular traps. Sci Immunol. 2018;3:eaar6689. 30143555 10.1126/sciimmunol.aar6689

[43] Chen KW, Monteleone M, Boucher D, Sollberger G, Ramnath D, Condon ND, et al. Noncanonical inflammasome signaling elicits gasdermin D-dependent neutrophil extracellular traps. Sci Immunol. 2018;3:eaar6676. 30143554 10.1126/sciimmunol.aar6676

[44] Kayagaki N, Stowe IB, Lee BL, O’Rourke K, Anderson K, Warming S, et al. Caspase-11 cleaves gasdermin D for non-canonical inflammasome signalling. Nature. 2015;526:666–71. 26375259 10.1038/nature15541

[45] Sarhan J, Liu BC, Muendlein HI, Li P, Nilson R, Tang AY, et al. Caspase-8 induces cleavage of gasdermin D to elicit pyroptosis during Yersinia infection. Proc Natl Acad Sci USA. 2018;115:E10888–E10897. 30381458 10.1073/pnas.1809548115 PMC6243247

[46] Demarco B, Grayczyk JP, Bjanes E, Le Roy D, Tonnus W, Assenmacher CA, et al. Caspase-8-dependent gasdermin D cleavage promotes antimicrobial defense but confers susceptibility to TNF-induced lethality. Sci Adv. 2020;6:eabc3465. 33208362 10.1126/sciadv.abc3465 PMC7673803

[47] Chen KW, Gross CJ, Sotomayor FV, Stacey KJ, Tschopp J, Sweet MJ, et al. The neutrophil NLRC4 inflammasome selectively promotes IL-1beta maturation without pyroptosis during acute Salmonella challenge. Cell Rep. 2014;8:570–82. 25043180 10.1016/j.celrep.2014.06.028

[48] Kambara H, Liu F, Zhang X, Liu P, Bajrami B, Teng Y, et al. Gasdermin D exerts anti-inflammatory effects by promoting neutrophil death. Cell Rep. 2018;22:2924–36. 29539421 10.1016/j.celrep.2018.02.067 PMC5878047

[49] Burgener SS, Leborgne NGF, Snipas SJ, Salvesen GS, Bird PI, Benarafa C. Cathepsin G inhibition by serpinb1 and Serpinb6 prevents programmed necrosis in neutrophils and monocytes and reduces GSDMD-driven inflammation. Cell Rep. 2019;27:3646–56 e3645. 31216481 10.1016/j.celrep.2019.05.065 PMC7350907

[50] He K, Wan T, Wang D, Hu J, Zhou T, Tao W, et al. Gasdermin D licenses MHCII induction to maintain food tolerance in small intestine. Cell. 2023;186:3033–48 e3020. 37327784 10.1016/j.cell.2023.05.027

[51] Zhang N, Zhang J, Yang Y, Shan H, Hou S, Fang H, et al. A palmitoylation-depalmitoylation relay spatiotemporally controls GSDMD activation in pyroptosis. Nat Cell Biol. 2024;26:757–69. 38538834 10.1038/s41556-024-01397-9

[52] Balasubramanian A, Hsu AY, Ghimire L, Tahir M, Devant P, Fontana P, et al. The palmitoylation of gasdermin D directs its membrane translocation and pore formation during pyroptosis. Sci Immunol. 2024;9:eadn1452. 38530158 10.1126/sciimmunol.adn1452 PMC11367861

[53] Zheng S, Que X, Wang S, Zhou Q, Xing X, Chen L, et al. ZDHHC5-mediated NLRP3 palmitoylation promotes NLRP3-NEK7 interaction and inflammasome activation. Mol Cell. 2023;83:4570–85 e4577. 38092000 10.1016/j.molcel.2023.11.015

[54] Yu T, Hou D, Zhao J, Lu X, Greentree WK, Zhao Q, et al. NLRP3 Cys126 palmitoylation by ZDHHC7 promotes inflammasome activation. Cell Rep. 2024;43:114070. 38583156 10.1016/j.celrep.2024.114070 PMC11130711

[55] Xu J, Pickard JM, Nunez G. FDA-approved disulfiram inhibits the NLRP3 inflammasome by regulating NLRP3 palmitoylation. Cell Rep. 2024;43:114609. 39116210 10.1016/j.celrep.2024.114609 PMC11398858

[56] Chu X, Zhang T, Bukhari I, Hu M, Xu J, Xing Y, et al. Ubiquitination of gasdermin D N-terminal domain directs its membrane translocation and pore formation during pyroptosis. Cell Death Dis. 2025;16:181. 40097387 10.1038/s41419-025-07475-6 PMC11914233

[57] Humphries F, Shmuel-Galia L, Ketelut-Carneiro N, Li S, Wang BW, Nemmara VV, et al. Succination inactivates gasdermin D and blocks pyroptosis. Science. 2020;369:1633. 32820063 10.1126/science.abb9818 PMC8744141

[58] Chu X, Xiao X, Wang G, Uosef A, Lou X, Arnold P, et al. Gasdermin D-mediated pyroptosis is regulated by AMPK-mediated phosphorylation in tumor cells. Cell Death Dis. 2023;14:469. 37495617 10.1038/s41419-023-06013-6 PMC10372026

[59] Xu W, Jin Q, Li X, Li D, Fu X, Chen N, et al. Crosstalk of HDAC4, PP1, and GSDMD in controlling pyroptosis. Cell Death Dis. 2024;15:115. 38326336 10.1038/s41419-024-06505-z PMC10850491

[60] Shi Y, Li X, Xu W, Wang Y, Dong L, Li D, et al. SUMOylation regulates GSDMD stability and pyroptosis. Int Immunopharmacol. 2025;149:114187. 39919454 10.1016/j.intimp.2025.114187

[61] Katoh M, Katoh M. Identification and characterization of human DFNA5L, mouse Dfna5l, and rat Dfna5l genes in silico. Int J Oncol. 2004;25:765–70. 15289881

[62] Yu C, Meng X, Zhang S, Zhao G, Hu L, Kong X. A 3-nucleotide deletion in the polypyrimidine tract of intron 7 of the DFNA5 gene causes nonsyndromic hearing impairment in a Chinese family. Genomics. 2003;82:575–9. 14559215 10.1016/s0888-7543(03)00175-7

[63] Rogers C, Fernandes-Alnemri T, Mayes L, Alnemri D, Cingolani G, Alnemri ES. Cleavage of DFNA5 by caspase-3 during apoptosis mediates progression to secondary necrotic/pyroptotic cell death. Nat Commun. 2017;8:14128. 28045099 10.1038/ncomms14128 PMC5216131

[64] Wang Y, Yin B, Li D, Wang G, Han X, Sun X. GSDME mediates caspase-3-dependent pyroptosis in gastric cancer. Biochem Biophys Res Commun. 2018;495:1418–25. 29183726 10.1016/j.bbrc.2017.11.156

[65] Hu Y, Liu Y, Zong L, Zhang W, Liu R, Xing Q, et al. The multifaceted roles of GSDME-mediated pyroptosis in cancer: therapeutic strategies and persisting obstacles. Cell Death Dis. 2023;14:836. 38104141 10.1038/s41419-023-06382-y PMC10725489

[66] Wang YP, Gao WQ, Shi XY, Ding JJ, Liu W, He HB, et al. Chemotherapy drugs induce pyroptosis through caspase-3 cleavage of a gasdermin. Nature. 2017;547:99. 28459430 10.1038/nature22393

[67] Tsuchiya K, Nakajima S, Hosojima S, Thi Nguyen D, Hattori T, Manh Le T, et al. Caspase-1 initiates apoptosis in the absence of gasdermin D. Nat Commun. 2019;10:2091. 31064994 10.1038/s41467-019-09753-2 PMC6505044

[68] Schneider KS, Gross CJ, Dreier RF, Saller BS, Mishra R, Gorka O, et al. The inflammasome drives GSDMD-independent secondary pyroptosis and IL-1 release in the absence of caspase-1 protease activity. Cell Rep. 2017;21:3846–59. 29281832 10.1016/j.celrep.2017.12.018 PMC5750195

[69] Chen KW, Demarco B, Ramos S, Heilig R, Goris M, Grayczyk JP, et al. RIPK1 activates distinct gasdermins in macrophages and neutrophils upon pathogen blockade of innate immune signaling. Proc Natl Acad Sci USA. 2021;118:e2101189118. 34260403 10.1073/pnas.2101189118 PMC8285957

[70] Zhang Z, Zhang Y, Xia S, Kong Q, Li S, Liu X, et al. Gasdermin E suppresses tumour growth by activating anti-tumour immunity. Nature. 2020;579:415–20. 32188940 10.1038/s41586-020-2071-9 PMC7123794

[71] Delmaghani S, del Castillo FJ, Michel V, Leibovici M, Aghaie A, Ron U, et al. Mutations in the gene encoding pejvakin, a newly identified protein of the afferent auditory pathway, cause DFNB59 auditory neuropathy. Nat Genet. 2006;38:770–8. 16804542 10.1038/ng1829

[72] Defourny J, Aghaie A, Perfettini I, Avan P, Delmaghani S, Petit C. Pejvakin-mediated pexophagy protects auditory hair cells against noise-induced damage. Proc Natl Acad Sci USA. 2019;116:8010–7. 30936319 10.1073/pnas.1821844116 PMC6475433

[73] Collin RW, Kalay E, Oostrik J, Caylan R, Wollnik B, Arslan S, et al. Involvement of DFNB59 mutations in autosomal recessive nonsyndromic hearing impairment. Hum Mutat. 2007;28:718–23. 17373699 10.1002/humu.20510

[74] Xia S, Zhang Z, Magupalli VG, Pablo JL, Dong Y, Vora SM, et al. Gasdermin D pore structure reveals preferential release of mature interleukin-1. Nature. 2021;593:607–11. 33883744 10.1038/s41586-021-03478-3 PMC8588876

[75] Liu Z, Wang C, Yang J, Zhou B, Yang R, Ramachandran R, et al. Crystal structures of the full-length murine and human gasdermin D reveal mechanisms of autoinhibition, lipid binding, and oligomerization. Immunity. 2019;51:43–49 e44. 31097341 10.1016/j.immuni.2019.04.017 PMC6640092

[76] Liu Z, Wang C, Rathkey JK, Yang J, Dubyak GR, Abbott DW, et al. Structures of the gasdermin D C-terminal domains reveal mechanisms of autoinhibition. Structure. 2018;26:778–84 e773. 29576317 10.1016/j.str.2018.03.002 PMC5932255

[77] Kuang S, Zheng J, Yang H, Li S, Duan S, Shen Y, et al. Structure insight of GSDMD reveals the basis of GSDMD autoinhibition in cell pyroptosis. Proc Natl Acad Sci USA. 2017;114:10642–7. 28928145 10.1073/pnas.1708194114 PMC5635896

[78] Evavold CL, Ruan J, Tan Y, Xia S, Wu H, Kagan JC. The pore-forming protein gasdermin D regulates interleukin-1 secretion from living macrophages. Immunity. 2018;48:35–44 e36. 29195811 10.1016/j.immuni.2017.11.013 PMC5773350

[79] Zhou ZD, Yi LX, Tan EK. Targeting gasdermin E in neurodegenerative diseases. Cell Rep Med. 2023;4:101075. 37343522 10.1016/j.xcrm.2023.101075 PMC10313929

[80] Pan J, Li Y, Gao W, Jiang Q, Geng L, Ding J, et al. Transcription factor Sp1 transcriptionally enhances GSDME expression for pyroptosis. Cell Death Dis. 2024;15:66. 38238307 10.1038/s41419-024-06455-6 PMC10796635

[81] Kayagaki N, Lee BL, Stowe IB, Kornfeld OS, O’Rourke K, Mirrashidi KM, et al. IRF2 transcriptionally induces GSDMD expression for pyroptosis. Sci Signal. 2019;12:eaax4917. 31113851 10.1126/scisignal.aax4917

[82] Wong WL, Su X, Li X, Cheung CM, Klein R, Cheng CY, et al. Global prevalence of age-related macular degeneration and disease burden projection for 2020 and 2040: a systematic review and meta-analysis. Lancet Glob Health. 2014;2:e106–116. 25104651 10.1016/S2214-109X(13)70145-1

[83] Owen CG, Jarrar Z, Wormald R, Cook DG, Fletcher AE, Rudnicka AR. The estimated prevalence and incidence of late stage age related macular degeneration in the UK. Br J Ophthalmol. 2012;96:752–6. 22329913 10.1136/bjophthalmol-2011-301109 PMC3329633

[84] Ambati J, Ambati BK, Yoo SH, Ianchulev S, Adamis AP. Age-related macular degeneration: etiology, pathogenesis, and therapeutic strategies. Surv Ophthalmol. 2003;48:257–93. 12745003 10.1016/s0039-6257(03)00030-4

[85] Ambati J, Fowler BJ. Mechanisms of age-related macular degeneration. Neuron. 2012;75:26–39. 22794258 10.1016/j.neuron.2012.06.018 PMC3404137

[86] Tarallo V, Hirano Y, Gelfand BD, Dridi S, Kerur N, Kim Y, et al. DICER1 loss and Alu RNA induce age-related macular degeneration via the NLRP3 inflammasome and MyD88. Cell. 2012;149:847–59. 22541070 10.1016/j.cell.2012.03.036 PMC3351582

[87] Wang SB, Narendran S, Hirahara S, Varshney A, Pereira F, Apicella I, et al. DDX17 is an essential mediator of sterile NLRC4 inflammasome activation by retrotransposon RNAs. Sci Immunol. 2021;6:eabi4493. 34860583 10.1126/sciimmunol.abi4493 PMC8767314

[88] Barnett KC, Liang K, Ting JP. Move over NAIP, DDX17 diversifies the NLRC4 inflammasome. Sci Immunol. 2021;6:eabm1201. 34860580 10.1126/sciimmunol.abm1201

[89] Gao J, Cui JZ, To E, Cao S, Matsubara JA. Evidence for the activation of pyroptotic and apoptotic pathways in RPE cells associated with NLRP3 inflammasome in the rodent eye. J Neuroinflammation. 2018;15:15. 29329580 10.1186/s12974-018-1062-3 PMC5766992

[90] Kerur N, Fukuda S, Banerjee D, Kim Y, Fu D, Apicella I, et al. cGAS drives noncanonical-inflammasome activation in age-related macular degeneration. Nat Med. 2018;24:50–61. 29176737 10.1038/nm.4450 PMC5760363

[91] Sekar R, Wooff Y, Cioanca AV, Kurera M, Ngo C, Man SM, et al. Impairing Gasdermin D-mediated pyroptosis is protective against retinal degeneration. J Neuroinflammation. 2023;20:239. 37864169 10.1186/s12974-023-02927-2 PMC10588253

[92] Cai B, Liao C, He D, Chen J, Han J, Lu J, et al. Gasdermin E mediates photoreceptor damage by all-trans-retinal in the mouse retina. J Biol Chem. 2022;298:101553. 34973334 10.1016/j.jbc.2021.101553 PMC8800116

[93] Gustavsson A, Norton N, Fast T, Frolich L, Georges J, Holzapfel D, et al. Global estimates on the number of persons across the Alzheimer’s disease continuum. Alzheimers Dement. 2023;19:658–70. 35652476 10.1002/alz.12694

[94] Palop JJ. Mucke L. Epilepsy and cognitive impairments in Alzheimer disease. Arch Neurol. 2009;66:435–40. 19204149 10.1001/archneurol.2009.15 PMC2812914

[95] Heneka MT, Carson MJ, El Khoury J, Landreth GE, Brosseron F, Feinstein DL, et al. Neuroinflammation in Alzheimer’s disease. Lancet Neurol. 2015;14:388–405. 25792098 10.1016/S1474-4422(15)70016-5 PMC5909703

[96] Rui W, Xiao H, Fan Y, Ma Z, Xiao M, Li S, et al. Systemic inflammasome activation and pyroptosis associate with the progression of amnestic mild cognitive impairment and Alzheimer’s disease. J Neuroinflammation. 2021;18:280. 34856990 10.1186/s12974-021-02329-2 PMC8638109

[97] Rui W, Wu Y, Yang Y, Xie W, Qin D, Ming J, et al. Myeloid gasdermin D drives early-stage T cell immunity and peripheral inflammation in a mouse model of Alzheimer’s disease. J Neuroinflammation. 2024;21:266. 39427168 10.1186/s12974-024-03255-9 PMC11491014

[98] Moonen S, Koper MJ, Van Schoor E, Schaeverbeke JM, Vandenberghe R, von Arnim CAF, et al. Pyroptosis in Alzheimer’s disease: cell type-specific activation in microglia, astrocytes and neurons. Acta Neuropathol. 2023;145:175–95. 36481964 10.1007/s00401-022-02528-y

[99] Wang Q, Guo S, Hu D, Dong X, Meng Z, Jiang Y, et al. Enhanced gasdermin-E-mediated pyroptosis in Alzheimer’s disease. Neuroscience. 2024;536:1–11. 37944579 10.1016/j.neuroscience.2023.11.004

[100] Taylor JP, Brown RH Jr, Cleveland DW. Decoding ALS: from genes to mechanism. Nature. 2016;539:197–206. 27830784 10.1038/nature20413 PMC5585017

[101] Van Schoor E, Ospitalieri S, Moonen S, Tom SO, Ronisz A, Ok O, et al. Increased pyroptosis activation in white matter microglia is associated with neuronal loss in ALS motor cortex. Acta Neuropathol. 2022;144:393–411. 35867112 10.1007/s00401-022-02466-9

[102] Cihankaya H, Bader V, Winklhofer KF, Vorgerd M, Matschke J, Stahlke S, et al. Elevated NLRP3 inflammasome activation is associated with motor neuron degeneration in ALS. Cells. 2024;13:995. 38920626 10.3390/cells13120995 PMC11202041

[103] Gunner G, Basu H, Lu Y, Bergstresser M, Neel D, Choi SY. et al. Gasdermin D is activated but does not drive neurodegeneration in SOD1 (G93A) model of ALS: Implications for targeting pyroptosis. Neurobiol Dis. 2025;214:107048 40759286 10.1016/j.nbd.2025.107048 PMC12412056

[104] Bates GP, Dorsey R, Gusella JF, Hayden MR, Kay C, Leavitt BR, et al. Huntington disease. Nat Rev Dis Primers. 2015;1:15005. 27188817 10.1038/nrdp.2015.5

[105] Crotti A, Glass CK. The choreography of neuroinflammation in Huntington’s disease. Trends Immunol. 2015;36:364–73. 26001312 10.1016/j.it.2015.04.007 PMC4786070

[106] Politis M, Lahiri N, Niccolini F, Su P, Wu K, Giannetti P, et al. Increased central microglial activation associated with peripheral cytokine levels in premanifest Huntington’s disease gene carriers. Neurobiol Dis. 2015;83:115–21. 26297319 10.1016/j.nbd.2015.08.011

[107] Bjorkqvist M, Wild EJ, Thiele J, Silvestroni A, Andre R, Lahiri N, et al. A novel pathogenic pathway of immune activation detectable before clinical onset in Huntington’s disease. J Exp Med. 2008;205:1869–77. 18625748 10.1084/jem.20080178 PMC2525598

[108] Paldino E, D'Angelo V, Laurenti D, Angeloni C, Sancesario G, Fusco FR. Modulation of inflammasome and pyroptosis by olaparib, a PARP-1 inhibitor, in the R6/2 mouse model of Huntingtonas disease. Cells. 2020;9:2286 33066292 10.3390/cells9102286 PMC7602058

[109] Ona VO, Li M, Vonsattel JP, Andrews LJ, Khan SQ, Chung WM, et al. Inhibition of caspase-1 slows disease progression in a mouse model of Huntington’s disease. Nature. 1999;399:263–7. 10353249 10.1038/20446

[110] Dendrou CA, Fugger L, Friese MA. Immunopathology of multiple sclerosis. Nat Rev Immunol. 2015;15:545–58. 26250739 10.1038/nri3871

[111] Pollock NM, Fernandes JP, Woodfield J, Moussa E, Hlavay B, Branton WG, et al. Gasdermin D activation in oligodendrocytes and microglia drives inflammatory demyelination in progressive multiple sclerosis. Brain Behav Immun. 2024;115:374–93. 37914099 10.1016/j.bbi.2023.10.022

[112] Li S, Wu Y, Yang D, Wu C, Ma C, Liu X, et al. Gasdermin D in peripheral myeloid cells drives neuroinflammation in experimental autoimmune encephalomyelitis. J Exp Med. 2019;216:2562–81. 31467036 10.1084/jem.20190377 PMC6829591

[113] Wang D, Zhang T, Shao Q, Wu X, Zhao X, Zhang H, et al. GSDME-mediated pyroptosis in microglia exacerbates demyelination and neuroinflammation in multiple sclerosis: insights from humans and cuprizone-induced demyelination model mice. Cell Death Differ. 2025;32:2368–2383. 40555745 10.1038/s41418-025-01537-0 PMC12669246

[114] Zhang X, Zhang Y, Wang B, Xie C, Wang J, Fang R, et al. Pyroptosis-mediator GSDMD promotes Parkinson’s disease pathology via microglial activation and dopaminergic neuronal death. Brain Behav Immun. 2024;119:129–45. 38552923 10.1016/j.bbi.2024.03.038

[115] Ma XX, Hao JN, Wu JR, Li YH, Cai XJ, Zheng YY. Prussian blue nanozyme as a pyroptosis inhibitor alleviates neurodegeneration. Adv Mater. 2022;34:e2106723. 35143076 10.1002/adma.202106723

[116] Wang B, Ma Y, Li S, Yao H, Gu M, Liu Y, et al. GSDMD in peripheral myeloid cells regulates microglial immune training and neuroinflammation in Parkinson’s disease. Acta Pharm Sin B. 2023;13:2663–79. 37425058 10.1016/j.apsb.2023.04.008 PMC10326292

[117] Wang M, Wang Y, Wang T, Han Y, Huang T, Du J, et al. Microglial GSDMD-mediated pyroptosis drives neuroinflammation in Parkinson’s disease. bioRxiv 2025.2006.2030.662314.

[118] Zhou B, Abbott DW. Chemical modulation of gasdermin D activity: therapeutic implications and consequences. Semin Immunol. 2023;70:101845. 37806032 10.1016/j.smim.2023.101845 PMC10841450

[119] Xiao J, Sun K, Wang C, Abu-Amer Y, Mbalaviele G. Compound loss of GSDMD and GSDME function is necessary to achieve maximal therapeutic effect in colitis. J Transl Autoimmun. 2022;5:100162. 36097634 10.1016/j.jtauto.2022.100162 PMC9463374

[120] Kopp A, Hagelueken G, Jamitzky I, Moecking J, Schiffelers LDJ, Schmidt FI, et al. Pyroptosis inhibiting nanobodies block Gasdermin D pore formation. Nat Commun. 2023;14:7923. 38040708 10.1038/s41467-023-43707-z PMC10692205

[121] Sun L, Wang H, Wang Z, He S, Chen S, Liao D, et al. Mixed lineage kinase domain-like protein mediates necrosis signaling downstream of RIP3 kinase. Cell. 2012;148:213–27. 22265413 10.1016/j.cell.2011.11.031

[122] Rathkey JK, Zhao J, Liu Z, Chen Y, Yang J, Kondolf HC, et al. Chemical disruption of the pyroptotic pore-forming protein gasdermin D inhibits inflammatory cell death and sepsis. Sci Immunol. 2018;3:eaat2738. 30143556 10.1126/sciimmunol.aat2738 PMC6462819

[123] Leem YH, Kim DY, Park JE, Kim HS. Necrosulfonamide exerts neuroprotective effect by inhibiting necroptosis, neuroinflammation, and alpha-synuclein oligomerization in a subacute MPTP mouse model of Parkinson’s disease. Sci Rep. 2023;13:8783. 37258791 10.1038/s41598-023-35975-y PMC10232437

[124] Jiao J, Wang Y, Ren P, Sun S, Wu M. Necrosulfonamide ameliorates neurological impairment in spinal cord injury by improving antioxidative capacity. Front Pharmacol. 2019;10:1538. 31998134 10.3389/fphar.2019.01538 PMC6962303

[125] Motawi TMK, Abdel-Nasser ZM, Shahin NN. Ameliorative effect of necrosulfonamide in a rat model of Alzheimer’s disease: targeting mixed lineage kinase domain-like protein-mediated necroptosis. ACS Chem Neurosci. 2020;11:3386–97. 32936609 10.1021/acschemneuro.0c00516

[126] Amara, Cooper N, Voronkova MA MP, Webb BA, Lynch EM, Kollman JM, et al. Selective activation of PFKL suppresses the phagocytic oxidative burst. Cell. 2021;184:4480–94 e4415. 34320407 10.1016/j.cell.2021.07.004 PMC8802628

[127] Cai W, Wu Z, Lai J, Yao J, Zeng Y, Fang Z, et al. LDC7559 inhibits microglial activation and GSDMD-dependent pyroptosis after subarachnoid hemorrhage. Front Immunol. 2023;14:1117310. 37063846 10.3389/fimmu.2023.1117310 PMC10090682

[128] Yu E, Zhang E, Lv X, Yan L, Lin Z, Siaw-Debrah F, et al. LDC7559 exerts neuroprotective effects by inhibiting GSDMD-dependent pyroptosis of microglia in mice with traumatic brain injury. J Neurotrauma. 2023;40:742–57. 35920115 10.1089/neu.2021.0318

[129] Campolo M, Casili G, Biundo F, Crupi R, Cordaro M, Cuzzocrea S, et al. The neuroprotective effect of dimethyl fumarate in an MPTP-mouse model of Parkinson’s disease: involvement of reactive oxygen species/nuclear factor-kappaB/nuclear transcription factor related to NF-E2. Antioxid Redox Signal. 2017;27:453–71. 28006954 10.1089/ars.2016.6800 PMC5564046

[130] Wang T, Sobue A, Watanabe S, Komine O, Saido TC, Saito T, et al. Dimethyl fumarate improves cognitive impairment and neuroinflammation in mice with Alzheimer’s disease. J Neuroinflammation. 2024;21:55. 38383481 10.1186/s12974-024-03046-2 PMC10882778

[131] Hu JJ, Liu X, Xia S, Zhang Z, Zhang Y, Zhao J, et al. FDA-approved disulfiram inhibits pyroptosis by blocking gasdermin D pore formation. Nat Immunol. 2020;21:736–45. 32367036 10.1038/s41590-020-0669-6 PMC7316630

[132] Wang C, Yang T, Xiao J, Xu C, Alippe Y, Sun K, et al. NLRP3 inflammasome activation triggers gasdermin D-independent inflammation. Sci Immunol. 2021;6:eabj3859. 34678046 10.1126/sciimmunol.abj3859 PMC8780201

[133] Zhang X, Huang X, Hang D, Jin J, Li S, Zhu Y, et al. Targeting pyroptosis with nanoparticles to alleviate neuroinflammatory for preventing secondary damage following traumatic brain injury. Sci Adv. 2024;10:eadj4260. 38198543 10.1126/sciadv.adj4260 PMC10780956

[134] Zhuang L, Luo X, Wu S, Lin Z, Zhang Y, Zhai Z, et al. Disulfiram alleviates pristane-induced lupus via inhibiting GSDMD-mediated pyroptosis. Cell Death Discov. 2022;8:379. 36057687 10.1038/s41420-022-01167-2 PMC9440918

[135] Mills EL, Ryan DG, Prag HA, Dikovskaya D, Menon D, Zaslona Z, et al. Itaconate is an anti-inflammatory metabolite that activates Nrf2 via alkylation of KEAP1. Nature. 2018;556:113–7. 29590092 10.1038/nature25986 PMC6047741

[136] Bambouskova M, Potuckova L, Paulenda T, Kerndl M, Mogilenko DA, Lizotte K, et al. Itaconate confers tolerance to late NLRP3 inflammasome activation. Cell Rep. 2021;34:108756. 33691097 10.1016/j.celrep.2021.108756 PMC8039864

[137] Bourner LA, Chung LA, Long H, McGettrick AF, Xiao J, Roth K, et al. Endogenously produced itaconate negatively regulates innate-driven cytokine production and drives global ubiquitination in human macrophages. Cell Rep. 2024;43:114570. 39093697 10.1016/j.celrep.2024.114570

[138] Xiong J, Lu DL, Chen BQ, Liu TY, Wang ZX. Dimethyl itaconate reduces cognitive impairment and neuroinflammation in APPswe/PS1DeltaE9 transgenic mouse model of Alzheimer’s disease. Neuromol Med. 2023;25:179–92. 10.1007/s12017-022-08725-y 35939256

[139] Kong X, Lyu W, Lin X, Lin C, Feng H, Xu L, et al. Itaconate alleviates anesthesia/surgery-induced cognitive impairment by activating a Nrf2-dependent anti-neuroinflammation and neurogenesis via gut-brain axis. J Neuroinflammation. 2024;21:104. 38649932 10.1186/s12974-024-03103-w PMC11034021

[140] Liu N, Jiang Y, Xiu Y, Tortelote GG, Xia W, Wang Y, et al. Itaconate restrains acute proinflammatory activation of microglia after traumatic brain injury in mice. Sci Transl Med. 2025;17:eadn2635. 40073156 10.1126/scitranslmed.adn2635

[141] Shan M, Zhang S, Luo Z, Deng S, Ran L, Zhou Q, et al. Itaconate promotes inflammatory responses in tissue-resident alveolar macrophages and exacerbates acute lung injury. Cell Metab. 2025;37:1750-1765.e7. 10.1016/j.cmet.2025.05.012 40527316

[142] Hu Y, Li H, Zhang X, Song Y, Liu J, Pu J, et al. Identification of two repurposed drugs targeting GSDMD oligomerization interface I to block pyroptosis. Cell Chem Biol. 2024;31:2024–38 e2027. 39486414 10.1016/j.chembiol.2024.10.002

[143] Schiffelers LDJ, Tesfamariam YM, Jenster LM, Diehl S, Binder SC, Normann S, et al. Antagonistic nanobodies implicate mechanism of GSDMD pore formation and potential therapeutic application. Nat Commun. 2024;15:8266. 39327452 10.1038/s41467-024-52110-1 PMC11427689

[144] Ma J, Xu J, Gao Q, Sun Y, Wang Y, Liu Z, et al. Engineering single-domain antibodies targeting Gasdermin E activation by the chemotherapeutic agent cis-diaminodichloroplatinum. Biotechnol J. 2023;18:e2200633. 37204010 10.1002/biot.202200633

[145] Wei C, Jiang W, Wang R, Zhong H, He H, Gao X, et al. Brain endothelial GSDMD activation mediates inflammatory BBB breakdown. Nature. 2024;629:893–900. 38632402 10.1038/s41586-024-07314-2

[146] Shi J, Zhao Y, Wang K, Shi X, Wang Y, Huang H, et al. Cleavage of GSDMD by inflammatory caspases determines pyroptotic cell death. Nature. 2015;526:660–5. 26375003 10.1038/nature15514

[147] Yang J, Liu Z, Wang C, Yang R, Rathkey JK, Pinkard OW, et al. Mechanism of gasdermin D recognition by inflammatory caspases and their inhibition by a gasdermin D-derived peptide inhibitor. Proc Natl Acad Sci USA. 2018;115:6792–7. 29891674 10.1073/pnas.1800562115 PMC6042100

[148] Wang P, Pan B, Tian J, Yang L, Chen Z, Yang L, et al. Ac-FLTD-CMK inhibits pyroptosis and exerts neuroprotective effect in a mice model of traumatic brain injury. NeuroReport. 2021;32:188–97. 33470761 10.1097/WNR.0000000000001580

[149] Schneider P, Walters WP, Plowright AT, Sieroka N, Listgarten J, Goodnow RA Jr, et al. Rethinking drug design in the artificial intelligence era. Nat Rev Drug Discov. 2020;19:353–64. 31801986 10.1038/s41573-019-0050-3

